# Identification of aberrant gene expression associated with aberrant promoter methylation in primordial germ cells between E13 and E16 rat F3 generation vinclozolin lineage

**DOI:** 10.1186/1471-2105-16-S18-S16

**Published:** 2015-12-09

**Authors:** Y-h Taguchi

**Affiliations:** 1 Department of Physics, Chuo University, Kasuga, Bunkyo-ku, 112-8551 Tokyo, Japan

**Keywords:** Transgenerational epigenetics, Principal component analysis, Feature extraction

## Abstract

**Background:**

Transgenerational epigenetics (TGE) are currently considered important in disease, but the mechanisms involved are not yet fully understood. TGE abnormalities expected to cause disease are likely to be initiated during development and to be mediated by aberrant gene expression associated with aberrant promoter methylation that is heritable between generations. However, because methylation is removed and then re-established during development, it is not easy to identify promoter methylation abnormalities by comparing normal lineages with those expected to exhibit TGE abnormalities.

**Methods:**

This study applied the recently proposed principal component analysis (PCA)-based unsupervised feature extraction to previously reported and publically available gene expression/promoter methylation profiles of rat primordial germ cells, between E13 and E16 of the F3 generation vinclozolin lineage that are expected to exhibit TGE abnormalities, to identify multiple genes that exhibited aberrant gene expression/promoter methylation during development.

**Results:**

The biological feasibility of the identified genes were tested via enrichment analyses of various biological concepts including pathway analysis, gene ontology terms and protein-protein interactions. All validations suggested superiority of the proposed method over three conventional and popular supervised methods that employed *t *test, limma and significance analysis of microarrays, respectively. The identified genes were globally related to tumors, the prostate, kidney, testis and the immune system and were previously reported to be related to various diseases caused by TGE.

**Conclusions:**

Among the genes reported by PCA-based unsupervised feature extraction, we propose that chemokine signaling pathways and leucine rich repeat proteins are key factors that initiate transgenerational epigenetic-mediated diseases, because multiple genes included in these two categories were identified in this study.

## Background

Transgenerational epigenetics (TGE) [1] describes the transfer of phenotypes between generations without the modification of genome sequences. Because the plant germline arises from somatic cells, TGE is often observed in plants. However, TGE was also reported in the offspring of mammals, when pregnant females are exposed to endocrine disruptions. Many factors are affected by TGE including male infertility [2], anxious behavior [3], mate preference [4], various diseases [5], reprogramming of primordial germ cells [6], and stress responses [7].

In contrast to reports studying the relationship of TGE to various abnormalities, few studies have investigated how TGE occurs. The main difficulty of studying TGE mechanisms is that epigenetic markers such as promoter methylation are not only heritable, but also vary over time during development in the generation associated with TGE. For example, for promoter methylation to affect development, it must be switched on/off during various stages of development [1]. Thus, TGE that affects development is expected to follow a similar time course. Therefore, abnormalities caused by TGE must be related to the aberrant timing of promoter methylation/demethylation when compared with normal organisms. Detecting small irregularities of promoter methylation timing based on comparisons with normal organisms is not easy. For example, Skinner et al [6] recently tried to identify aberrant gene expression associated with aberrant promoter methylation between E13 and E16 germ line in F3 generation vinclozolin lineages, where vinclozolin functions as an endocrine disruptor. Endocrine disruption is thought to cause various diseases especially in reproductive organs, because it is often misrecognized as a hormone effect on the development of reproductive organs. Thus, usage of endocrine disruptors is usually forbidden for public health. Furthermore, vinclozolin was recently observed to cause TGE abnormalities. However, Skinner et al failed to identify strict pairs of aberrant gene expression and promoter methylation for specific genes. They concluded "A comparison between the germ cell DMR (differential DNA methylated regions) and the differentially expressed genes indicated no significant overlap". Thus, our understanding of the mechanisms by which TGE occurs remains poor.

In the present study we applied the recently proposed principal component analysis (PCA)-based unsupervised feature extraction (FE) [8-17] to the data set obtained by Skinner et al [6] and successfully identified a significant overlap between DMR and differentially expressed genes. Various methods for enrichment analyses supported the biological feasibility of the 48 identified RefSeq mRNAs. We also confirmed the superiority of the proposed methodology over three other methods. The relatively poorer performances achieved by these three methodologies compared with PCA-based unsupervised FE indicated that the proposed methodology outperformed these three frequently employed methods.

Furthermore, 22 genes among those derived from the 48 RefSeq mRNAs identified by PCA-based unsupervised FE were previously reported to be related to diseases caused by TGE [5]. This suggests that aberrant gene expression associated with aberrant promoter methylation during this stage of development is a key factor in the generation of TGE-mediated diseases. Because multiple genes involved in chemokine signaling pathways or containing leucine-rich repeat (LRR) proteins were identified in the current study, we hypothesized that chemokine signaling pathways and/or LRR proteins were involved in mediating TGE-related diseases.

### Previous usage of PCA-based unsupervised FE

Here, we briefly review previous studies [8-17] that used PCA-based unsupervised FE. In Refs. [8-11], we applied PCA-based unsupervised FE to microRNA expression for biomarker identification between patients (of various diseases including various cancers, chronic obstructive pulmonary disease, and Alzheimer's disease, etc) and healthy controls; microRNA extracted in an unsupervised manner was combined with linear discriminant analysis. We found a combination of 10-20 microRNAs generally achieved about 80 % accuracy. It was also confirmed that the identified set of microRNAs were stable. Thus, this method is robust for the selection of samples. In Ref. [12], we applied PCA-based unsupervised FE to the proteome in a bacterial culture and identified critical proteins in an unsupervised manner. In Ref. [13], we applied PCA-based unsupervised FE to mRNA and miRNA expression of stressed mouse heart. After identifying potential disease causing genes, we performed *in silico *drug discovery of the identified genes. In Ref. [14], we performed integrated analysis of promoter methylation profiles of three distinct autoimmune diseases using PCA-based unsupervised FE and identified many genes commonly associated with aberrant promoter methylation. In Ref. [15], we applied PCA-based unsupervised FE to genotyping/DNA methylation profiles of cancer and identified genotype specific DNA methylation profiles that occurred in cancer genetics. In Refs [16,17], PCA-based unsupervised FE of mRNA expression and promoter methylation profiles of normal/treated cancer cell lines was investigated. Based upon the integrated analysis of mRNA expression and promoter methylation profiles, we identified potential disease causing genes.

In summary, PCA-based unsupervised FE has mainly been used to compare between patients (or cancer cell lines) and healthy controls excluding one exception [12]. Because it is likely that healthy controls and patients (or control and treated cancer cell lines) exhibit distinct expressions, it is not surprising that PCA-based unsupervised FE detected significant differences, even if most of the biomarker/disease causing genes were identified only by PCA-based unsupervised FE, but not by other methodologies. In this study, we applied PCA-based unsupervised FE to a different factor, the difference between two time points (E13 and E16). These time points represent different developmental stages and thus some differences are expected; however, the time points are separated by only 3 days, and therefore the differences should be much smaller than between healthy controls and patients (or control and treated cancer cell lines). Of note, although Skinner et al [6] reported no aberrant gene expression associated with aberrant promoter methylation between E13 and E16 germ lines in F3 generation vinclozolin lineages, the study was still published. Thus, from a methodological point of view, the purpose of this study was to investigate whether PCA-based unsupervised FE could identify slight differences; thus it is a new challenge for this methodology.

## Methods

### Gene expression and promoter methylation profiles

Gene expression/promoter methylation profiles were retrieved from the gene expression omnibus (GEO) using GEO ID GSE59511. This super series consists of two subseries, GSE43559 and GSE59510, each of which includes gene expression (using Affymetrix Rat Gene 1.0 ST Array) and promoter methylation (using NimbleGen Rat CpG Island Plus RefSeq Promoter 720k array) information, respectively. Gene expression profiles were directly loaded from GEO to R [18] by getGEO function while six files whose names ended with ratio_peaks_mapToFeatures_All_Peaks.txt.gz were downloaded and loaded into R using read.csv for promoter methylation. Table 1 shows a list of the samples analyzed. GSE43559 (gene expression) consists of eight samples classified into four categories, E13 control, E13 treated, E16 control, and E16 treated. GSE59510 (promoter methylation) consists of six samples classified into two categories, E13 and E16 (all from F3 generation primordial germ lines). Using the ratio between treated and control groups, eight gene expression profiles were converted to alternative eight profiles as follows:

**Table 1:** Gene expression and promoter methylation profiles.

GEO ID	Description
GSE43559 (gene expression)	

GSM1065332	PGC E13 F3-Control biological rep1
GSM1065333	PGC E13 F3-Control biological rep2
GSM1065334	PGC E13 F3-Vinclozolin biological rep1
GSM1065335	PGC E13 F3-Vinclozolin biological rep2
GSM1065336	PGC E16 F3-Control biological rep1
GSM1065337	PGC E16 F3-Control biological rep2
GSM1065338	PGC E16 F3-Vinclozolin biological rep1
GSM1065339	PGC E16 F3-Vinclozolin biological rep2

GSE59510 (promoter methylation)	

GSM1438556	E16-Vip2/Cip2
GSM1438557	E13-Vip2/Cip1
GSM1438558	E13-Vip1/Cip1
GSM1438559	E16-Vip1/Cip1
GSM1438560	E16-Vip2/Cip1
GSM1438561	E13-Vip2/Cip2

$$\left(\begin{array}{c} \frac{\text{E}13\;\text{Control}\;\text{rep}1}{\text{E}13\;\text{treated}\;\text{rep}1}\\ \frac{\text{E}13\;\text{Control}\;\text{rep}2}{\text{E}13\;\text{treated}\;\text{rep}2}\\ \frac{\text{E}13\;\text{Control}\;\text{rep}2}{\text{E}13\;\text{treated}\;\text{rep}1}\\ \frac{\text{E}13\;\text{Control}\;\text{rep}1}{\text{E}13\;\text{treated}\;\text{rep}2}\\ \frac{\text{E}16\;\text{Control}\;\text{rep}1}{\text{E}16\;\text{treated}\;\text{rep}1}\\ \frac{\text{E}16\;\text{Control}\;\text{rep}2}{\text{E}16\;\text{treated}\;\text{rep}2}\\ \frac{\text{E}16\;\text{Control}\;\text{rep}2}{\text{E}16\;\text{treated}\;\text{rep}1}\\ \frac{\text{E}16\;\text{Control}\;\text{rep}1}{\text{E}16\;\text{treated}\;\text{rep}2} \end{array}\right).$$

The reason we used a ratio of control to treated instead of the usual ratio of treated to control is explained in additional file 1. These were further normalized to have a mean of zero and a variance of one within each sample. Because six samples in GSE59510 were already transformed to a ratio between treated/control samples, these were not normalized. In total, 14 (8+6) samples that exhibited a ratio between control/treated samples were pooled and prepared for further analyses. The only difference between control and treated samples was whether oil or vinclozolin was injected to F1 pregnant rats between E8 and E14. Any other treatments were identical between E13 and E16.

### Principal component analysis-based unsupervised feature extraction

Although this method was described in detail in a recently published review article [19], this methodology is briefly introduced here. Example: *x_ij _*is the gene expression/promoter methylation of the *i*th gene (*i *= 1, ..., *N*) in the *j*th sample (*j *= 1, ..., *M*). For simplicity, it is assumed that the mean of *x_ij _*over *i *within each *j *is zero. Then, in contrast to the ordinary usage of PCA where samples are embedded into the low dimensional space, genes are embedded into the low dimensional space by applying PCA. Thus, principal component (PC) scores of the *ℓ*th component, *x_iℓ_*, (*ℓ *= 1, ..., *M*) are attributed to each gene while each sample has contributed *c_ℓj _*to the *ℓ*th component. By this definition, *x_iℓ _*is expressed as

$$x_{i\ell }=\underset{j}{\sum }c_{\ell j}x_{ij}$$

PCA-based unsupervised FE attempts to extract features (in this specific application, genes) with larger absolute PC scores along the specified *ℓ*th PC.

In the specific application described in the present study, $N_{\text{expression}}^{\prime }$ probes using gene expression and $N_{\text{methylation}}^{\prime }$ probes using promoter methylation were selected, respectively. For the computation of *P*-values of coincident analysis with binomial distribution, $N_{\text{expression}}^{\prime }=N_{\text{methylation}}^{\prime }=N^{\prime }$ for simplicity.

Although there are several ways to determine which PC is employed for FE, the most straightforward and intuitive strategy is to identify PCs that are mostly coincident with categories by employing categorical regression:

$$c_{\ell j}=a_{\ell }+\underset{k}{\sum }a_{k\ell }\delta_{kj}$$

where *a_ℓ _*and *a_kℓ _*are numerical (regression) coefficients. Then, the *ℓ*th PC associated with the (most) significant regression is employed as the PC for FE. Because this study only contained two categories (E13 and E16), we used the *t *test instead of categorical regression to measure the significance of coincidence between *c_ℓj _*and categories.

### *t *test-based FE

The *t *test was applied to gene expression/promoter methylation in each probe. For gene expression, $\left(\begin{array}{c} \frac{\text{E}13\;\text{Control}\;\text{rep}1}{\text{E}13\;\text{treated}\;\text{rep}1}\\ \frac{\text{E}13\;\text{Control}\;\text{rep}2}{\text{E}13\;\text{treated}\;\text{rep}2}\\ \frac{\text{E}13\;\text{Control}\;\text{rep}2}{\text{E}13\;\text{treated}\;\text{rep}1}\\ \frac{\text{E}13\;\text{Control}\;\text{rep}1}{\text{E}13\;\text{treated}\;\text{rep}2} \end{array}\right)$ and $\left(\begin{array}{c} \frac{\text{E}16\;\text{Control}\;\text{rep}1}{\text{E}16\;\text{treated}\;\text{rep}1}\\ \frac{\text{E}16\;\text{Control}\;\text{rep}2}{\text{E}16\;\text{treated}\;\text{rep}2}\\ \frac{\text{E}16\;\text{Control}\;\text{rep}2}{\text{E}16\;\text{treated}\;\text{rep}1}\\ \frac{\text{E}16\;\text{Control}\;\text{rep}1}{\text{E}16\;\text{treated}\;\text{rep}2} \end{array}\right)$ were compared. For promoter methylation, $\left(\begin{array}{c} \frac{\text{E}13\text{ - Vip}2}{\text{E}13\text{ - Cip}1}\\ \frac{\text{E}13\text{ - Vip}1}{\text{E}13\text{ - Cip}1}\\ \frac{\text{E}13\text{ - Vip}2}{\text{E}13\text{ - Cip}2} \end{array}\right)$ and $\left(\begin{array}{c} \frac{\text{E}16\text{ - Vip}2}{\text{E}16\text{ - Cip}1}\\ \frac{\text{E}16\text{ - Vip}1}{\text{E}16\text{ - Cip}1}\\ \frac{\text{E}16\text{ - Vip}2}{\text{E}16\text{ - Cip}2} \end{array}\right)$ were compared. Then the most significant (i.e., associated with smaller *P*-values) $N_{\text{expression}}^{\prime }$ and $N_{\text{methylation}}^{\prime }$ probes were selected, respectively. For the computation of *P*-values of coincident analysis with binomial distribution, $N_{\text{expression}}^{\prime }=N_{\text{methylation}}^{\prime }=N^{\prime }$ for simplicity.

### limma-based FE

limma [20] was applied to gene expression and promoter methylation as follows. For gene expression, after converting raw gene expression to logarithmic values, the model Diff = (E16.VIN-E16.CNTL)-(E13.VIN-E16.CNTL) was applied, where VIN and CNTL correspond to treated and control samples, respectively. For promoter methylation, only the ratio between control and treated samples was provided (see Table 1), and the two class model was applied for E13 and E16 samples (R source codes are shown in additional file 1). Then the obtained *P*-values were employed for FE. The remaining procedures were the same as for the previous two FEs.

### SAM-based FE

SAM [21] was applied to gene expression and promoter methylation separately, as shown in Table 1, i.e., as two class problems of E13 and E16 (siggenes packages [22] in Bioconductor [23] was used). Then, the obtained *P*-values were used for FE. The remaining procedures were the same as for the previous three FEs.

### Protein-protein interaction enrichment analysis

The obtained RefSeq mRNA IDs were converted to gene names ("official gene symbol") via a gene ID conversion tool implemented in DAVID [24], and the obtained gene names were uploaded to STRING [25] server. Then, "protein-protein interactions" was selected among the pull-down menu of "enrichment", where the expected number of PPIs for the set of genes uploaded and the *P*-value attributed to identified PPIs are available.

### Gene ID identification for literature searches

Literature searches were performed using gene symbols that were converted from RefSeq mRNAs using DAVID as explained above.

## Results and Discussion

### Gene selection using PCA-based unsupervised FE

**Figure 1:**
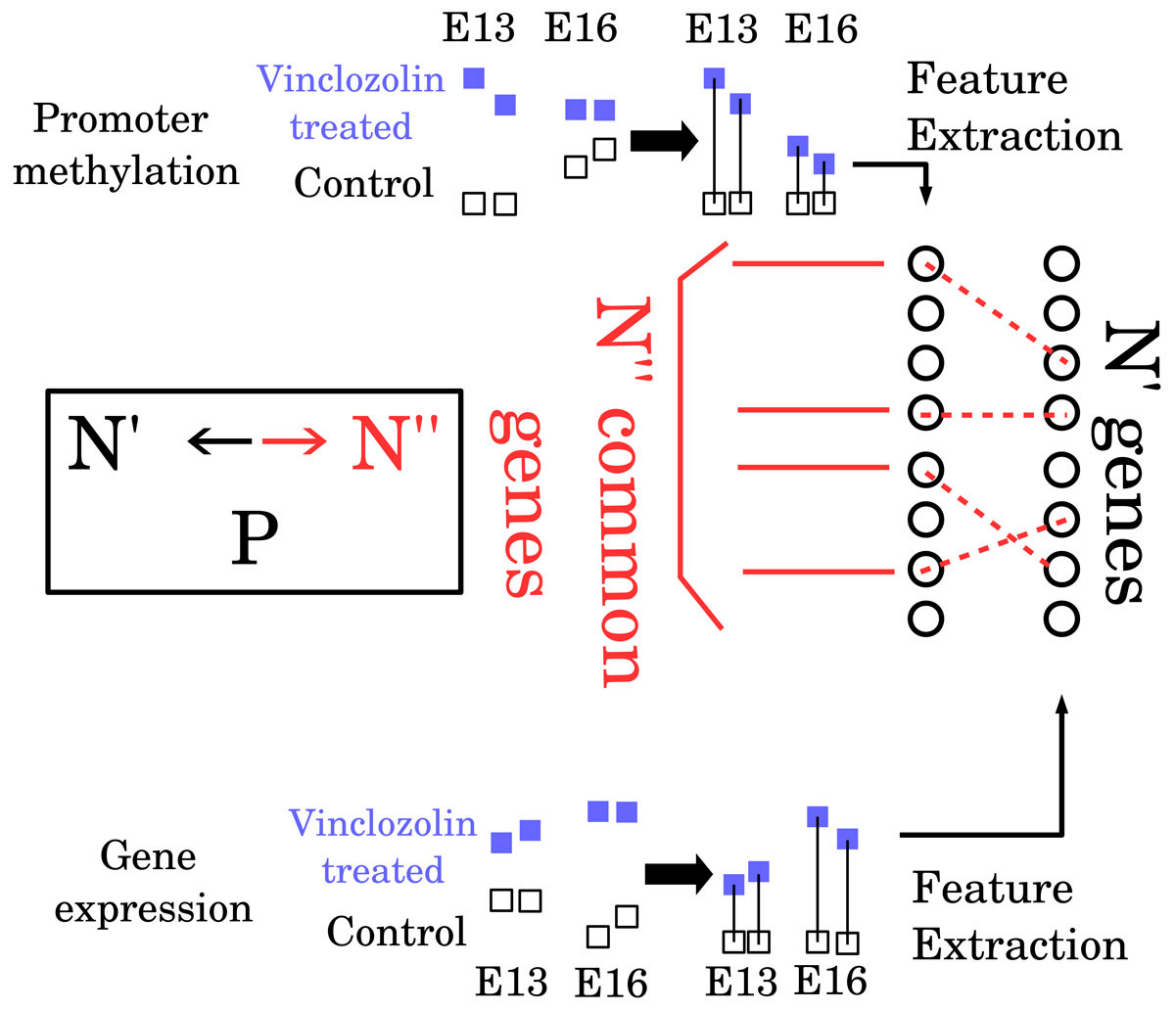
**Schematics that illustrate the procedure of PCA-based unsupervised FE applied to data set analyzed in the present study**.

Figure 1 illustrates the strategy to identify aberrant gene expression associated with aberrant promoter methylation between controls and vinclozolin treated samples during development from E13 to E16. Gene expression and promoter methylation of vinclozolin treated F3 samples were normalized relative to controls. Then, by separately applying PCA-based unsupervised FE to each sample group, the top *N' *(*≪ N*) genes were independently selected. The number of commonly selected genes *N'' *was counted. If *N'' *was much larger than expected, the selection of aberrant gene expression associated with aberrant promoter methylation was determined to be successful.

**Figure 2:**
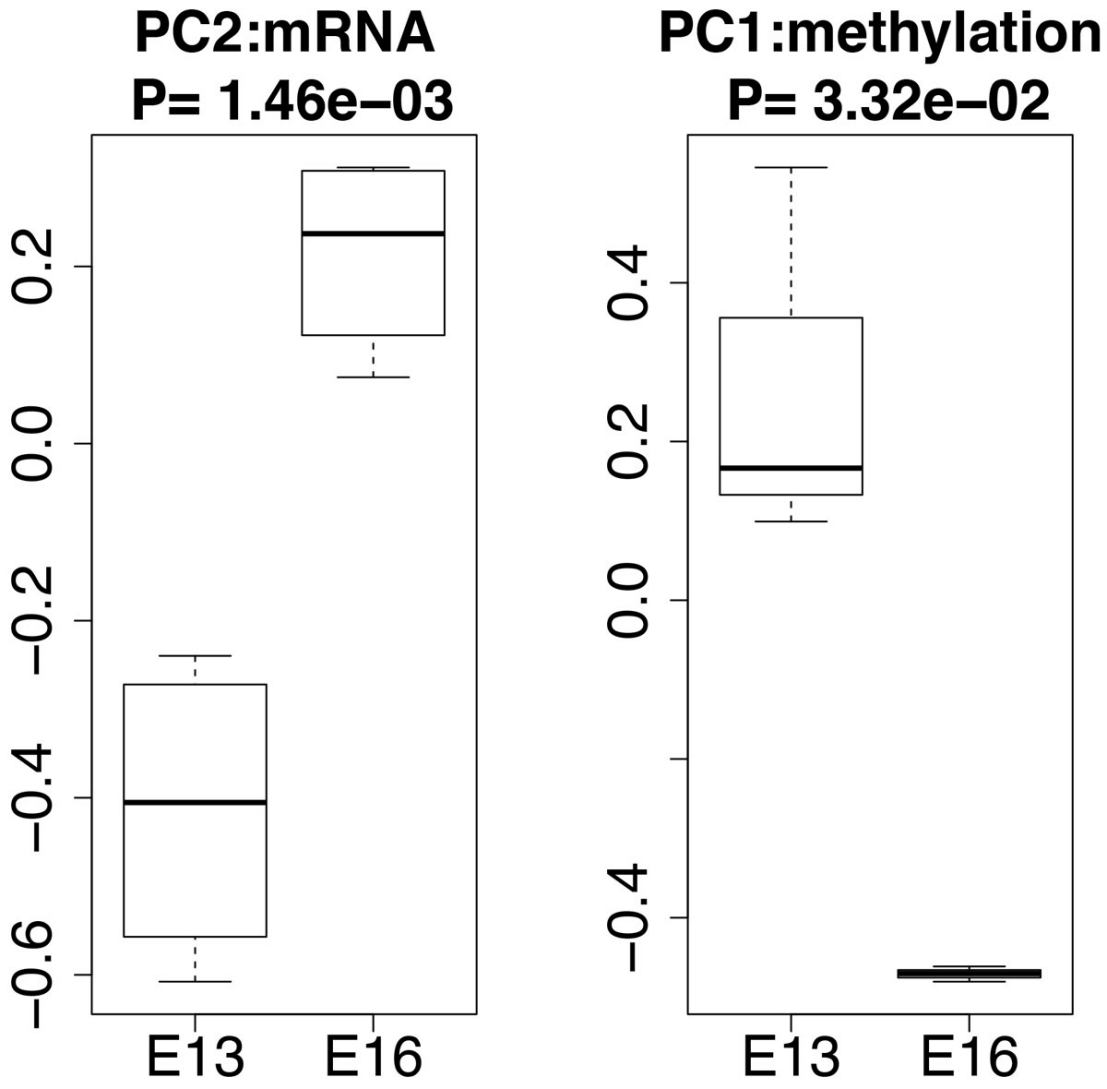
**Boxplots of PCs used for FE in this study, PC2 for mRNA and PC1 for methylation**. *P*-values are computed by *t *test.

**Figure 3:**
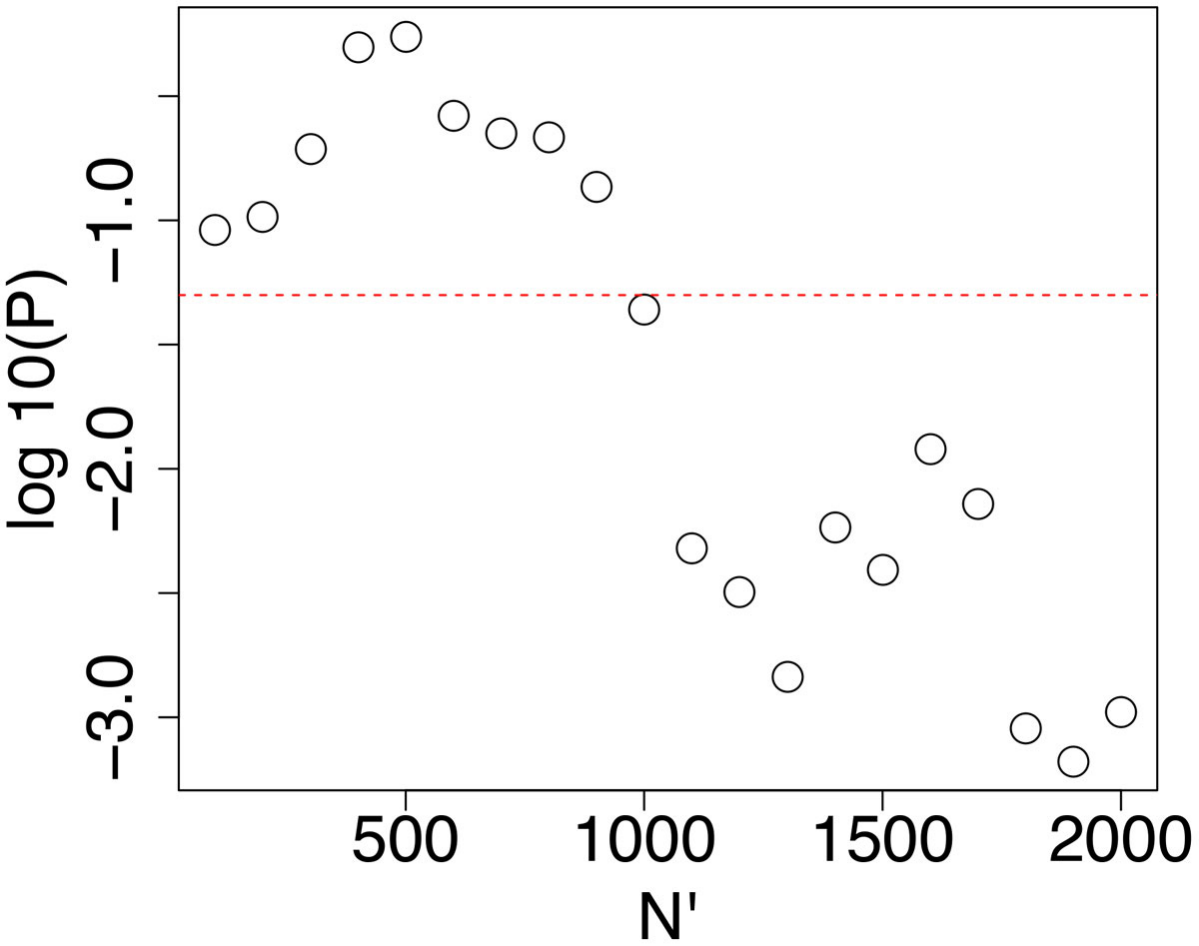
**Dependence of logarithmic *P*-values that represent the significance of commonly selected genes between gene expression and promoter methylation upon *N' *when PCA-based unsupervised FE was employed**. Horizontal broken red line represents *P *= 0.05.

At first, the PCs used for FE shown in Figure 1 were specified and a boxplot (PC2 for mRNA and PC1 for methylation) is shown in Figure 2. These two PCs exhibited a significant distinction between the two categories, E13 and E16. Using the specified PCs, PCA-based unsupervised FE was performed. Then, the most significant *N' *genes were extracted for gene expression and promoter methylation, respectively. *P*-values to determine whether the coincidence and the number of commonly selected genes among *N' *genes occurred accidentally was computed by binomial distribution. How the *P*-values varied dependent upon *N' *was determined. Figure 3 shows the dependence of *P*-values upon *N' *when *N *= 13324, the number of genes commonly included in gene expression and promoter methylation profiles. *P*-values were smaller for larger *N'*. However, the minimum *N' *with *P*-values less than 0.05 were selected (i.e., *N' *= 1000) to minimize the number of genes selected to reduce the time spent performing literature searches in the later part of this study. Among the 1000 genes selected in either gene expression or promoter methylation, 48 RefSeq mRNAs were commonly selected (a list of gene names and boxplots of individual genes are shown in additional files 2 and 3). The *P*-value for *N' *= 1000 was 0.04 (see Figure 3, and this value was confirmed by the shuffle test, additional file 1). Thus, we successfully selected genes that were significantly associated with simultaneous aberrant gene expression/promoter methylation.

To biologically validate these 48 RefSeq mRNAs, we uploaded them to three enrichment analyses servers, DAVID [24], TargetMine [26] and g:Profiler [27]. We observed some biological terms were enriched among the selected genes (Table 2). Almost 50% of the genes selected belonged to G-protein coupled receptors (GPCR) or cell surface receptor pathways, which was expected because an endocrine disruptor such as vinclozolin targets cell surface receptors. We also estimated PPI enrichment (see methods). Because it is rare for proteins to function in the absence of collaboration with other proteins, enriched PPIs among the selected genes (proteins) can provide supporting evidence for the biological significance of selected genes. There were seven PPIs although the expected number of PPIs was three. This resulted in *P *= 0.05; thus there was significant PPI enrichment among the genes selected by PCA-based unsupervised FE.

**Table 2:** Enrichment analysis of 48 RefSeq mRNAs commonly selected in the top most 1000 genes by applying PCA-based unsupervised FE to gene expression and promoter methylation.

DAVID			
GO BP			
GO:0007186	19	G-protein coupled receptor protein signaling pathway	5.35E-03
GO:0007166	21	Cell surface receptor linked signal transduction	4.19E-03
g:proflier			
GO BP			
GO:0003008	17	System process	4.37E-02
GO:0007166	22	Cell surface receptor signaling pathway	8.91E-03
GO MF			
GO:0060089	17	Molecular transducer activity	4.49E-02
GO:0004871	17	Signal transducer activity	1.82E-02
GO:0004872	17	Receptor activity	1.13E-02
GO:0038023	17	Signaling receptor activity	3.98E-03
GO:0004888	16	Transmembrane signaling receptor activity	1.08E-02
GO:0004930	14	G-protein coupled receptor activity	4.43E-02

*P*-values shown in Figure 3 remained significant even when *N' *increased from 1000 to 2000. Thus, we tried to obtain more genes by setting *N' *= 2000, because the greater number of genes uploaded would have a tendency to enhance enrichment. There were 179 mRNAs commonly selected between gene expression and promoter methylation (see additional file 3 for the full list). Uploading these genes to three enrichment analyses servers resulted in greater enrichment for these 179 genes as expected (Tables 3, 4, and 5).

**Table 3:** Enrichment analysis of 179 genes commonly selected in the top most 2000 genes by applying PCA-based unsupervised FE to gene expression and promoter methylation.

DAVID KEGG			
rno04740	50	Olfactory transduction	1.63E-15
GO BP			
GO:0007186	79	G-protein coupled receptor protein signaling pathway	2.04E-20
GO:0007166	85	Cell surface receptor linked signal transduction	2.39E-18
GO:0050911	59	Detection of chemical stimulus involved in sensory perception of smell	1.99E-18
GO:0050907	59	Detection of chemical stimulus involved in sensory perception	2.22E-18
GO:0009593	59	Detection of chemical stimulus	3.09E-18
GO:0007608	59	Sensory perception of smell	3.38E-18
GO:0050906	59	Detection of stimulus involved in sensory perception	3.26E-18
GO:0007606	60	Sensory perception of chemical stimulus	2.89E-18
GO:0051606	60	Detection of stimulus	2.88E-18
GO:0007600	61	Sensory perception	3.31E-16
GO:0050890	62	Cognition	2.44E-15
GO:0050877	62	Neurological system process	1.94E-12
GO CC			
GO:0016021	101	Integral to membrane	3.57E-12
GO:0031224	101	Intrinsic to membrane	1.65E-11
GO:0031983	7	Vesicle lumen	1.49E-03
GO:0060205	6	Cytoplasmic membrane-bounded vesicle lumen	7.41E-03
GO:0031091	6	Platelet alpha granule	1.59E-02
GO:0031093	5	Platelet alpha granule lumen	3.82E-02
GO MF			
GO:0004984	60	Olfactory receptor activity	1.59E-19

**Table 4:** Enrichment analysis of 179 genes commonly selected in the top most 2000 genes by applying PCA-based unsupervised FE to gene expression and promoter methylation.

g:profiler GO BP			
GO:0007606	54	Sensory perception of chemical stimulus	9.14E-21
GO:0007186	65	G-protein coupled receptor signaling pathway	7.61E-20
GO:0050911	50	Detection of chemical stimulus involved in sensory perception of smell	1.44E-19
GO:0007600	58	Sensory perception	2.89E-19
GO:0050907	50	Detection of chemical stimulus involved in sensory perception	5.26E-19
GO:0007608	50	Sensory perception of smell	5.65E-19
GO:0009593	50	Detection of chemical stimulus	1.72E-18
GO:0050906	50	Detection of stimulus involved in sensory perception	3.39E-18
GO:0007166	84	Cell surface receptor signaling pathway	4.19E-18
GO:0003008	69	System process	8.92E-18
GO:0051606	51	Detection of stimulus	1.26E-17
GO:0050877	59	Neurological system process	3.82E-16
GO:0051716	106	Cellular response to stimulus	6.09E-13
GO:0042221	84	Response to chemical	9.54E-13
GO:0050896	116	Response to stimulus	4.65E-12
GO:0007154	98	Cell communication	4.91E-12
GO:0007165	92	Signal transduction	2.84E-11
GO:0044700	95	Single organism signaling	6.05E-11
GO:0023052	95	Signaling	6.70E-11
GO:0065007	131	Biological regulation	3.40E-10
GO:0050789	128	Regulation of biological process	3.48E-10
GO:0050794	120	Regulation of cellular process	1.92E-07
GO:0044707	94	Single-multicellular organism process	9.54E-07
GO:0032501	94	Multicellular organismal process	8.75E-06
GO:0044763	129	Single-organism cellular process	1.17E-05
GO:0044699	135	Single-organism process	1.86E-04
GO:0046010	3	Positive regulation of circadian sleep/wake cycle, non-REM sleep	2.21E-02
GO CC			
GO:0016021	88	Integral component of membrane	1.13E-12
GO:0031224	88	Intrinsic component of membrane	3.85E-12
GO:0071944	79	Cell periphery	1.19E-08
GO:0044425	92	Membrane part	1.43E-08
GO:0005886	77	Plasma membrane	3.24E-08
GO:0016020	97	Membrane	1.09E-02
GO MF			
GO:0038023	70	Signaling receptor activity	5.11E-023
GO:0004930	64	G-protein coupled receptor activity	5.42E-023
GO:0004888	68	Transmembrane signaling receptor activity	1.3E-022
GO:0004871	72	Signal transducer activity	1E-021
GO:0004872	70	Receptor activity	4.63E-021
GO:0060089	72	Molecular transducer activity	5.95E-020
GO:0004984	50	Olfactory receptor activity	1.39E-019
KEGG			
KEGG:04740	42	Olfactory transduction	6.46E-014
KEGG:05144	5	Malaria	1.96E-02

**Table 5:** Enrichment analysis of 179 genes commonly selected in the top most 2000 genes by applying PCA-based unsupervised FE to gene expression and promoter methylation.

TargetMine GO BP			
GO:0007600	43	Sensory perception	5.81E-12
GO:0007606	40	Sensory perception of chemical stimulus	8.20E-12
GO:0050907	38	Detection of chemical stimulus involved in sensory perception	2.64E-11
GO:0051606	39	Detection of stimulus	2.64E-11
GO:0009593	38	Detection of chemical stimulus	3.56E-11
GO:0050906	38	Detection of stimulus involved in sensory perception	3.60E-11
GO:0003008	48	System process	3.93E-11
GO:0050877	43	Neurological system process	5.27E-11
GO:0007186	41	G-protein coupled receptor signaling pathway	3.63E-09
GO:0007166	46	Cell surface receptor signaling pathway	2.01E-06
GO:0042221	59	Response to chemical	3.63E-06
GO:0044707	60	Single-multicellular organism process	3.94E-05
GO:0032501	61	Multicellular organismal process	6.44E-05
GO:0050911	24	Detection of chemical stimulus involved in sensory perception of smell	9.90E-05
GO:0007608	24	Sensory perception of smell	1.24E-04
GO:0051716	59	Cellular response to stimulus	1.04E-03
GO:0007165	49	Signal transduction	1.30E-03
GO:0050896	68	Response to stimulus	2.64E-03
GO:0065007	75	Biological regulation	4.11E-03
GO:0007154	52	Cell communication	4.97E-03
GO:0050789	71	Regulation of biological process	1.07E-02
GO:0023052	49	Signaling	2.56E-02
GO:0044700	49	Single organism signaling	2.56E-02
GO:0044699	84	Single-organism process	4.23E-02
GO CC			
GO:0016021	46	Integral component of membrane	8.14E-07
GO:0031224	46	Intrinsic component of membrane	9.96E-07
GO:0044425	51	Membrane part	3.43E-04
GO:0016020	56	Membrane	2.37E-02
GO MF			
GO:0004871	45	Signal transducer activity	5.80E-10
GO:0004888	43	Transmembrane signaling receptor activity	5.80E-10
GO:0038023	44	Signaling receptor activity	5.80E-10
GO:0004872	44	Receptor activity	7.10E-10
GO:0060089	45	Molecular transducer activity	7.10E-10
GO:0004984	24	Olfactory receptor activity	8.31E-05
KEGG			
rno04740	50	Olfactory transduction	1.05E-13

GPCR and cell surface receptors were enhanced and olfactory transduction related biological terms were vastly enriched. Careful investigation of the selected genes indicated that many olfactory receptor proteins were newly identified when *N' *was increased from 1000 to 2000. Olfactory receptor proteins were also recognized by Skinner et al [6]. Thus, the identification of many olfactory receptor proteins suggested the correctness and superiority of our methodology, because Skinner et al [6] did not identify reciprocal relationships between gene expression and promoter methylation, probably owing to a lack of suitable statistical methods, although they noted their importance.

PPI enrichment significance was also enhanced when *N' *increased from 1000 to 2000. There were 360 PPIs among 179 genes while the expected number of PPIs was 191. This resulted in *P *= 0 (within the numerical accuracy adopted); thus the significance of PPI enrichment was enhanced. The increase of PPIs was mostly due to the newly identified olfactory receptor proteins.

These data suggest the biological suitability of our methodology.

### Comparisons with other supervised FEs

Although PCA-based unsupervised FE was already demonstrated to perform better than various conventional methods reported for various applications [8-17], other simpler methods might achieve a comparative performance in this specific example, although the study by Skinner et al [6] was unsuccessful. To demonstrate the superiority of PCA-based unsupervised FE compared with a simpler method, genes were selected by *t *test between E13 and E16. The *t *test was applied to the F3 generation vinclozolin lineage gene expression/promoter methylation and normalized to control samples (see methods) and the most *N' *significant genes were selected for both gene expression and promoter methylation. Then the significance of overlap between genes selected by the *t *test related to gene expression and promoter methylation was determined. Results demonstrated that in the range of *N' ≤ *2000, the minimum achieved *P *was 0.38, which was not significant (additional file 4). *P*-values increased as *N' *approached 2000 in contrast to the tendency seen in Figure 3 and there were no overlaps when *N' ≤ *300; thus, the *P*-value could not be computed. Therefore, in this specific example, PCA-based unsupervised FE achieved a good performance compared with a simpler method. The second method we compared was limma [20] (see Methods), a popular method used for gene expression analyses, especially when genes exhibit differential expression between multiple conditions. In the first page of the manual, limma was identified as aiming to analyze a small number of samples; "Empirical Bayesian methods are used to provide stable results even when the number of arrays is small". Thus, limma is a very suitable method whose performance should be compared with PCA based unsupervised FE that also aims to treat small sample cases. The results were disappointing. Within the range of *N' ≤ *2000, there were only two *N' *associated with *P*-values less than 0.05, when *N'*s tested were taken to be equivalent to those when PCA-based unsupervised FE as well as *t *test-based FE were employed (additional file 4). The number of genes selected commonly between gene expression and promoter methylation was also small. When *N' *= 800 such that there were as many common genes as possible, the number of genes commonly selected between gene expression and promoter methylation was 33 Refseq mRNAs, among which there were no overlaps with the 48 RefSeq mRNAs selected by PCA-based unsupervised FE when *N' *= 1000 (the list of 33 RefSeq mRNAs identified by limma-based FE is shown in additional file 3). Furthermore, biological validation was also disappointing. Even uploading these 33 RefSeq mRNAs to three enrichment servers, the identified enrichments were zero. DAVID, g:Profiler or TargetMine identified no enriched GO BP, CC, MF terms or KEGG pathways. There were also no PPIs detected by STRING among the RefSeq mRNAs genes. Moreover, because there are no larger *N' *associated with *P*-values less than 0.05, we could not increase the number of common genes such that more enrichments were detected. The third method we compared was SAM [21] (see Methods), another popular method used for gene expression analyses, also designed for multiclass problems. The results were again disappointing. Within the range of *N' ≤ *2000, there were only four *N' *associated with *P*-values less than 0.05, when *N′*s tested were taken to be equivalent to those when the other three methods were employed (additional file 4). The number of genes selected commonly between gene expression and promoter methylation was also small. When *N' *= 800 such that there were as many common genes as possible, the number of genes commonly selected between gene expression and promoter methylation was 30 RefSeq mRNAs (the list of 30 RefSeq mRNAs identified by SAM-based FE is shown in additional file 3), among which there were 11 RefSeq mRNAs that were also included in the 48 RefSeq mRNAs identified by PCA-based unsupervised FE when *N' *= 1000. Furthermore, biological validation was also disappointing. No GO BP, CC, MF terms or KEGG pathway enrichments were identified when these 30 RefSeq mRNAs were uploaded to the DAVID, g:Profiler or TargetMine enrichment servers. There were also only two PPIs (*P *= 0.37, thus not significant) detected by STRING among the RefSeq mRNAs genes. Moreover as for limma, because there are no larger *N' *associated with *P*-values less than 0.05, we could not increase the number of common genes such that more enrichments were detected.

Thus, we conclude that PCA-based unsupervised FE outperformed simple *t *test-based FE, the sophisticated Bayesian methodology (limma)-based FE, and the popular SAM-based FE. Therefore, the superiority of PCA-based unsupervised FE to these methods was demonstrated.

### Biological significance of selected genes: literature searches

To estimate the biological significance of the selected 48 RefSeq mRNAs selected by PCA-based unsupervised FE when *N' *= 1000 in more detail, we performed a literature search for each gene selected. During the searches, we focused on the relationship between selected genes and diseases; Anway et al [5] reported that TGE induced by vinclozolin caused tumor, prostate, kidney, testis and immune diseases. Table 6 summarizes the association of selected genes previously reported to be related to tumor, prostate, kidney, testis and immune disease. Of the 48 RefSeq mRNAs identified, 22 were associated with targeted properties (the list of references identified by literature searches as well as detailed discussions are available in additional file 1). This indicates the success of our methodology to identify genes potentially associated with causing TGE mediated diseases in the F3 generation vinclozolin lineage.

**Table 6:** Summary of literature searches for genes selected by PCA-based unsupervised FE when *N' *= 1000.

Gene	Tumors	Prostate	Kidney	Testis	Immune Disease
CCR2	○	○	○		○
LRRN3	○				○
AHR	○	○	○	○	○
LOX	○	○	○	○	○
PRAMEL1	○	○		○	
CD53	○		○		○
ITGAL (CD11A)	○	○	○	○	○
SULT1C2			○		
FCGR2B			○		○
ELOVL2				○	
PF4 (CXCL4)	○	○	○	○	
PDHA2				○	
MPO	○	○	○	○	○
HAND2	○				
CCL3	○	○		○	○
CMKLR1 (CHEMR23)			○	○	○
DBH	○	○	○	○	○
KCNT1			○		
FGB	○	○	○		○
BMP3	○	○	○		
ACTG2			○		
AQP2	○	○	○		

Circles indicate that there was at least one study reporting a relationship between identified gene and disease. For related references, see additional file 1. Genes that were reported to be related to tumor, prostate, kidney, testis and immune diseases are listed.

To further compare genes selected by PCA-based unsupervised FE with those selected by limma and SAM from a biological point of view, we also performed literature searches of the 33 RefSeq mRNAs selected by limma (Table 7) and 19 RefSeq mRNAs selected by SAM but not included in the 48 Refseq mRNAs identified by PCA-based unsupervised FE when *N' *= 1000 (Table 8). The list of references identified by literature searches is shown in additional file 1. The limma-based FE was inferior to PCA-based unsupervised FE because only 13 genes were identified by the literature search and were reported to be related to lower numbers of terms including "tumors", "prostate", "kidney", "testis" and "immune". On the other hand, the SAM-based FE showed more promising results, which was expected because it identified 11 RefSeq mRNAs that overlapped with 48 RefSeq mRNAs identified by PCA-based unsupervised FE when *N' *= 1000. In Table 8, 11 out of 19 genes were associated with disease. Therefore, it might be thought that SAM-based FE was superior to PCA-based unsupervised FE, which only identified 22 disease associated genes among 48 genes. However, PCA-based unsupervised FE also identified 179 genes by increasing *N' *from 1000 to 2000, which is impossible for SAM-based FE. We found that only three genes (IL15, PGAM2, and ZFP36L1) that were not included in the 179 RefSeq mRNAs identified by PCA-based unsupervised FE when *N' *= 2000. This suggested that PCA-based unsupervised FE identified almost the same genes as cover those identified by SAM-based FE. Although the disease associations of genes identified by PCA-based unsupervised FE and previously reported in the literature might not have been confirmed or remain just an observation or hypothesis, the strict difference in disease associated genes identified by PCA-based unsupervised FE and those by other methods suggested the superiority of PCAbased unsupervised FE.

**Table 7:** Summary of literature searches for genes selected by limma-based FE.

Gene	Tumors	Prostate	Kidney	Testis	Immune Disease
qk	○	○		○	
dpf1					○
TOP1	○	○			
Arhgef1	○				
TEAD2	○				
Sirt2	○	○	○		
gmfg	○				
alkbh6	○				
MCEE	○				
hbs1l	○				
HSPBP1	○				
XRCC1	○	○	○	○	

Circles indicate that there was at least one study reporting a relationship. For related references, see additional file 1. Genes that were reported to be related to tumor, prostate, kidney, testis and immune diseases are listed.

**Table 8:** Summary of literature searches for genes selected by SAM-based FE, but not by PCA-based unsupervised FE when *N' *= 1000.

Gene	Tumors	Prostate	Kidney	Testis	Immune Disease
MYL1	○				
SLC28A1	○		○		
PGAM2	○			○	
Alb	○	○	○	○	○
SLC13A3	○	○	○		
TTR	○	○	○	○	○
ANGPTL1	○				
TUBB3	○	○	○	○	
IL15	○	○	○	○	○
BATCh1	○	○	○		
ZFP36L1	○				○

Circles indicate that there was at least one study reporting a relationship. For related references, see additional file 1. Genes that were reported to be related to tumor, prostate, kidney, testis and immune diseases are listed.

Thus, PCA-based unsupervised FE appeared superior to limma and SAM-based FE.

### Two groups of selected genes: chemokine signaling pathway genes and LRR proteins

To provide further insights of the genes selected by PCA-based unsupervised FE when *N' *= 1000, we focused on two categories: chemokine signaling pathway genes and LRR proteins, both of which have been extensively reported to be related to vinclozolin mediated diseases. This also supports the effectiveness of our methodology and the importance of the selected genes.

#### Chemokine signaling pathway

Four chemokine/chemokine receptors were selected: CCR2, PF4 (CXCL4), CCL3, and CMKLR1. The first three belong to the chemokine signaling pathway (rno04062) in KEGG, as either ligands or receptors that activate chemokine signaling pathways. In addition, CMKLR1 is a chemokine receptor-like protein although it is not included in the KEGG pathway. They are all localized at cell surfaces, and therefore are expected to function together to activate/inactivate chemokine signaling pathways. It is reasonable that they are detected together in the present analysis. Some studies also suggested a relationship between chemokines and vinclozolin. Cowin et al [28] reported that prostatic inflammation was associated with the postpubertal activation of proinflammatory NF*κ*B-dependent genes, including the chemokine IL-8 when embryos were exposed to vinclozolin in rats. Chemokine signaling pathways were associated with genes whose expression was altered in rat F3 generation vinclozolin lineage Sertoli cells [2]. The expression of Cxcr4 and Cxcl2 was altered in vinclozolin F3 generation rat prostate epithelial cells [28]. Interestingly, together with Cxcr4 and Cxcl2, BMP6 was reported to have an altered expression [28]. BMP6 shares binding specificity with BMP3, which was also identified in this study (see Table 6). BMP proteins belong to the TGF*β *pathway (rno04350) that is grouped with the chemokine signaling pathway as part of the cytokine-cytokine receptor interaction system (rno04060). Furthermore, numerous studies have suggested a relationship between chemokine signaling pathways and various diseases. For example, the inhibition of chemokine-induced biological activity is a promising therapeutic strategy for proteinuric disorders [29]. Chemokines and chemokine receptors play critical roles in prostate cancer development and progression [30] as well as in testicular inflammation [31]. Thus, TGE abnormalities caused by vinclozolin might develop through the regulation of inherited promoter methylation during the stages of development, by affecting chemokine signaling pathways.

#### LRR proteins

LRRN3, PRAMEL1, and LRRTM1 are LRR proteins shown in Table 6 or in additional files 2 and 3. LRR proteins were frequently reported to be associated with F3 generation vinclozolin lineages, e.g., LRRc48, LRRc56, and LRRc8B [2], LR-RTM3 and ELFN2 [3], Lrfn3 [28], Lrrc46, Lrrc48, Lgi2, Fbxl7, Lgi4, Lrig1, Fbxl12, Lrfn1, Lingo1, Lrrc8b, Lrrn2, and LRRTM1. Lrrtm4 [4], Lrrc61 [32], and Lrrc56 [6] were reported to be related to F3 generation vinclozolin lineages. Although the frequent observation of aberrant LRR protein expression in F3 generation vinclozolin lineages does not always indicate that LRR proteins are potential causative factors of vinclozolin mediated transgenerational epigenetic-induced diseases, there are multiple reports that suggest a relationship between LRR proteins and nervous system disorders. LRRTM1, LRRTM3, LRRN1 and LRRN3 were reported to be related to autism spectrum disorder [33] and polymorphisms in LRR genes are associated with autism spectrum disorder susceptibility in populations of European ancestry [33]. Deletions in the LRRTM binding partner neurexin 1 (NRXN1) have also been linked to schizophrenia [34]. Moreover, LRR protein dysfunction may disrupt neuronal excitation/inhibition balance and contribute to neuropsychiatric disorders [35,36]. LRRTM3 was also identified as a candidate gene for late-onset Alzheimer's disease [37]. Toll-like receptors, transmembrane LRR proteins that bind a wide molecular variety of pathogen-associated ligands and are involved in immune responses and have been implicated in neurodegenerative diseases such as multiple sclerosis, stroke, and Alzheimer's disease [38,39]. Moreover, LRR proteins are generally believed to play critical roles in the development of neural circuits [35,36]; e.g., LRRTMs and neuroligins bind neurexins differentially to cooperate in glutamate synapse development [40]. Although neuroligins and neurexins mediate connections between pre/post-synapses [41], LRR proteins co-function with neuroligins and neurexins [36]. However, there are multiple reports that suggest vinclozolin mediates nervous system disorders. For example, the exposure of rats to vinclozolin increased risk for autism [7]. Furthermore, perinatal exposure to endocrine disruptors was generally associated with autism spectrum disorder [42] and exposure to vinclozolin significantly increased vulnerability to anxiety [43]. Thus, this association was suggested to be heritable [44]. Therefore, it is not surprising that LRR proteins cause vinclozolin mediated neuropsychiatric disorders including autism spectrum disorder.

## Conclusions

This study re-analyzed the gene expression/promoter methylation profiles of primordial germ cells between E13 and E16 rat F3 generation vinclozolin lineage [6]. In contrast to analyses performed previously [6], we successfully identified various genes associated with aberrant promoter methylation/gene expression using treated and control samples. Identified genes were related to previously reported diseases in F3 generation vinclozolin lineage. We focused on two categories, chemokine signaling pathway molecules and LRR proteins, that might be disease causing factors. The success of the study methodology suggests the possibility that abnormalities in F3 generation vinclozolin lineage are mediated by heritable aberrant promoter methylation during development between generations.

## Competing interests

The authors declare that they have no competing interests.

## Authors' contributions

YHT planned and performed all analyses and wrote the paper.

## Supplementary Material

Additional file 1 **Supplementary discussions**. Click here for file

Additional file 2 **List of genes selected by PCA-based unsupervised FE, limma-based FE, and SAM-based FE**. Click here for file

Additional file 3 Boxplots for the 48 genes identified by PCA-based unsupervised FE when *N' *= 1000. Click here for file

Additional file 4 Dependence of *P *values upon *N' *when genes are selected by the *t *test, limma and SAM instead of PCA-based unsupervised FE. Click here for file

## Declarations

Publication charges for this article have been funded by Chuo University Joint Research Grant.

This article has been published as part of *BMC Bioinformatics *Volume 16 Supplement 18, 2015: Joint 26th Genome Informatics Workshop and 14th International Conference on Bioinformatics: Bioinformatics. The full contents of the supplement are available online at http://www.biomedcentral.com/bmcbioinformatics/supplements/16/S18.
