# Supplementary material for: Identification of aberrant gene expression associated with aberrant promoter methylation in primordial germ cells between E13 and E16 rat F3 generation vinclozolin lineage
Source: BMC Bioinformatics. 2015 Dec 9;16(Suppl 18):S16. doi: 10.1186/1471-2105-16-S18-S16 (PMC4682393; doi:10.1186/1471-2105-16-S18-S16)
Supplement: Additional file 1 — Supplementary discussions. [file 1471-2105-16-S18-S16-S1.pdf]

Supplementary Document

Y-h. Taguchi, Department of Physics, Chuo University, Tokyo, Japan

Contents

1 Reason for the usage of control/treated instead of treated/control 2

2 Replacing binomial test with shuffle test 2

3 R codes for limma executions 2

4 Biological significance of selected genes: literature searches 3
4.1 Genes identified by PCA based unsupervised FE when $N' = 1000$ . . . 3
4.1.1 CCR2 . . . 3
4.1.2 LRRN3 . . . 3
4.1.3 AHR . . . 3
4.1.4 LOX . . . 3
4.1.5 PRAMEL1 . . . 3
4.1.6 CD53 . . . 3
4.1.7 ITGAL . . . 4
4.1.8 SULT1C2 . . . 4
4.1.9 FCGR2B . . . 4
4.1.10 ELOVL2 . . . 4
4.1.11 PF4 . . . 4
4.1.12 PDHA2 . . . 4
4.1.13 MPO . . . 4
4.1.14 HAND2 . . . 4
4.1.15 CCL3 . . . 4
4.1.16 HBE2 . . . 5
4.1.17 CMKLR1 . . . 5
4.1.18 DBH . . . 5
4.1.19 KCNT1 . . . 5
4.1.20 TAAR7B . . . 5
4.1.21 Fibrinogen beta chain . . . 5
4.1.22 BMP3 . . . 5
4.1.23 ACTG2 . . . 5
4.1.24 AQP2 . . . 5
4.2 Genes identified by limma based FE . . . 6
4.2.1 qk . . . 6
4.2.2 TOP1 . . . 6
4.2.3 Arhgef1 . . . 6
4.2.4 TEAD2 . . . 6
4.2.5 Sirt2 . . . 6
4.2.6 gmfg . . . 6
4.2.7 alkbh6 . . . 6
4.2.8 MCEE . . . 6
4.2.9 hbs1l . . . 6
4.2.10 HSPBP1 . . . 6
4.2.11 XRCC1 . . . 6
4.3 Genes identified by SAM based FE, but not by PCA based unsupervised FE, when $N' = 1000$ . . . 7
4.3.1 MYL1 . . . 7
4.3.2 SLC28A1 . . . 7
4.3.3 PGAM2 . . . 7
4.3.4 ALB . . . 7
4.3.5 SLC13A3 . . . 7
4.3.6 TTR . . . 7

4.3.7 ANGPTL1 7
4.3.8 TUBB3 7
4.3.9 IL15 7
4.3.10 BACH1 7
4.3.11 ZFP36L2 7

1 Reason for the usage of control/treated instead of treated/control

One may wonder why the ratio control/treated instead of treated/control was used for gene expression, because the latter is more commonly used. The primary purpose of PCA based unsupervised FE is the identification of outliers. Thus, no matter how the ratio is defined, outliers are expected to remain outliers. If treated/control was employed, 44 genes instead of 48 genes are commonly selected between gene expression and promoter methylation if $N' = 1000$. Thus, the number of commonly selected genes did not change drastically. In addition, among 44 genes, 42 genes are selected when control/treated was employed. Thus, genes commonly selected between gene expression and promoter methylation did not alter dependent upon whether control/treated instead of treated/control was used for gene expression. Because more common genes indicate a more feasible methodology, we used ratio control/treated instead of treated/control.

2 Replacing binomial test with shuffle test

One may wonder if the usage of binomial test to estimate P-value for commonly selected genes is reasonable. To validate usage of the binomial test, we shuffled gene order within gene expression/promoter methylation profiles independently one thousand times and repeated the whole analyses with $N' = 1000$. We found that the frequency of commonly selected genes was greater than 48, which is the number of commonly selected genes in the original (unshuffled) data set, and was 36 among one thousand trials, which is fully coincident with a probability of 0.04 estimated by binomial test. This definitely supports the usage of binomial test.

3 R codes for limma executions

For gene expression (eight samples shown in Table 1 of main text), suppose that `x` includes not a ratio but raw gene expression (rows are probes and columns are samples) with the first column of probe ids,

```
TS <-c(rep("E13.CNTL",2),rep("E13.VIN",2),rep("E16.CNTL",2),rep("E16.VIN",2))
require(Biobase)
require(limma)
gene_exp <- new("ExpressionSet",expr=data.matrix(log(x[,-1])))
fData(gene_exp)[["gene_id"]] <- x[,1]
pData(gene_exp)[["sample_name"]] <- TS
TS <- factor(TS)
design <- model.matrix(~0+TS)
colnames(design) <- levels(TS)
fit <- lmFit(gene_exp, design)
cont.matrix <- makeContrasts(Diff=(E16.VIN-E16.CNTL)-(E13.VIN-E16.CNTL),levels=design)
fit2 <- contrasts.fit(fit, cont.matrix)
fit2 <- eBayes(fit2)
TT <- topTable(fit2,number=27342)
```

For promoter methylation (six samples shown in Table 1 of main text), suppose that `xm` includes promoter methylation (rows are probes and columns are samples) with the first column of probe ids,

```
xm[is.na(xm)] <- 1
gene_exp <- new("ExpressionSet",expr=data.matrix(log(xm[,-1])))
fData(gene_exp)[["gene_id"]] <- xm[,1]
pData(gene_exp)[["sample_name"]] <- c(rep("E13",3),rep("E16",3))
design <- cbind(WT=1,class=c(0,0,0,1,1,1))
fit <- lmFit(gene_exp, design)
```

```
fit <- eBayes(fit)
TT1 <- topTable(fit, coef="class",number=14162)
```

4 Biological significance of selected genes: literature searches

4.1 Genes identified by PCA based unsupervised FE when $N' = 1000$

4.1.1 CCR2

CC chemokine receptor type-2 (CCR2) is a member of the G-protein coupled receptor superfamily, and is expressed on the cell surface of monocytes and macrophages. It binds to monocyte chemoattractant protein-1, a CC chemokine, produced at the sites of inflammation and infection. Based on their roles in disease, they are attractive targets for the pharmaceutical industry, and thus, targeting both CCR2 and CCR5 can be a useful strategy [1]. Blockade of CCR2 ameliorates progressive fibrosis in the kidney [2]. TR4 nuclear receptor promotes prostate cancer metastasis via the upregulation of CCL2/CCR2 signaling [3]. CCR2 is related to cancer immunotherapy [4].

4.1.2 LRRN3

3D structures of some LRR protein–ligand complexes show that the concave surface of the LRR domain is ideal for interaction with α-helix, indicating that the elongated and curved LRR structure provides a framework for achieving diverse protein–protein interactions [5]. LRRN3 exhibits aberrant expression in neuroblastoma [6]. LRRN3 was suggested to be a novel, clinically relevant biomarker of immune status in HIV-1 infection [7].

4.1.3 AHR

AHR is a ligand-activated transcription factor that controls the expression of a diverse set of genes [8]. One of its known ligands is dioxin, a highly toxic environmental pollutant that is similar to vinclozolin. Vinclozolin was shown to be a weak inducer of AHR [9], which was suggested to be a therapeutic target for chronic kidney disease [10]. AHR has critical roles in sperm development [11]. AHR-mediated genotoxic effects were observed in a prostate cancer cell line [12]. AHR was previously reported to target multiple genes suggested to participate in non-small-cell lung cancer metastasis [13]. AHR also plays critical roles in immunology [14].

4.1.4 LOX

Lysyl oxidase (LOX) is a copper-dependent amine oxidase with a critical role in the biogenesis of connective tissue matrices by crosslinking the extracellular matrix proteins, collagen and elastin. LOX was upregulated in kidney abscesses [15] and affects prostate cancer growth [16]. LOX knockout mice develop sexual development problems including reduced production of sperm [17]. LOX-mediated collagen crosslinking is responsible for fibrosis-enhanced metastasis [18], and LOX plays critical roles in mesenchymal stem cell-driven breast cancer malignancy [19]. LOX is generally recognized to be a critical factor of tumor genesis and is targeted for cancer treatment [20].

4.1.5 PRAMEL1

PRAMEL1 is a member of the melanoma antigen preferentially expressed in tumors (PRAME) gene family. The PRAME gene family encodes LRR proteins that function as transcription regulators in cancer cells [21]. The only normal tissues that expressed PRAME proteins were the testes [22]. The aberrant methylation of PRAMEL1 was observed in Dnmt1 overexpressing prostate tissue of mice [23].

4.1.6 CD53

The interaction of mesangial cells with the extracellular matrix plays a major role in kidney biology. CD53 is present in mesangial cells *in vivo* and in culture [24]. Natural killer (NK) cells are important contributors to the early immune defense against infected or transformed cells. The tetraspanin CD53 modulates responses from activating NK cell receptors, promoting lymphocyte function-associated antigen activation and dampening NK cell effector functions [25]. Polymorphonuclear neutrophils in systemic lupus erythematosus, an autoimmune disease, exhibit the enhanced expression of CD53 [26]. CD53, together with carcinoembryonic antigen-related cell adhesion molecule 1, was targeted by early B cell factor (EBF)-1 [27].

4.1.7 ITGAL

ITGAL, also known as CD11A, is one of six genes that is used to successfully discriminate patients with castration-resistant prostate cancer into two risk groups [28]. ITGAL together with CD18 involved in the leukocyte adhesion pathway plays a role in mediating ischemic acute renal failure in rats [29]. CD11A was shown to be absent in testis tumors [30] but was essential for inflammatory and immune responses [31]. ITGAL was previously also reported to be associated with another F3 generation vinclozolin lineage [32].

4.1.8 SULT1C2

SULT1C2 was shown to be abundant in the kidney but was absent in a model of kidney disease [33].

4.1.9 FCGR2B

FCGR2B and FCRLB gene polymorphisms are associated with IgA nephropathy (kidney disease) [34]. FCGR2B expression on B cells and dendritic cells is important for the mucosal induction of antigen-specific immune tolerance [35].

4.1.10 ELOVL2

ELOVL2 controls the level of n-6 28:5 and 30:5 fatty acids in the testes [36]. EVOVL2 was previously reported to be associated with another F3 generation vinclozolin lineage [37].

4.1.11 PF4

Platelet factor 4 (PF4), also known as chemokine (C-X-C motif) ligand 4 (CXCL4), is a small cytokine belonging to the CXC chemokine family. PF4 has a protective role in chronic kidney allografts [38]. Pf4/Cxcl4 was upregulated in the testes of 5-day old rats of 2,3,7,8-tetrachlorodibenzo-p-dioxin-treated dams [39]. PF4 progresses prostate cancer [40] but down-regulates CC chemokine receptors, including CCR2 in human monocytes [41].

4.1.12 PDHA2

PDHA2 is one subunit of the pyruvate dehydrogenase complex [42] and is testis specific gene [43]. The activation of PDHA2 gene expression during spermatogenesis is thought to ensure the continued expression of the protein, thus allowing germ cell viability and functionality [44]. The selection of PDHA2 in this study that integrated gene expression and promoter methylation is convincing because the expression of PDHA2 is mediated by promoter methylation [45].

4.1.13 MPO

Myeloperoxidase (MPO) is a peroxidase enzyme related to renal diseases [46, 47], upregulated in ischemia-reperfusion testes [48], expressed in the prostate gland [49], associated with prostate cancer [50], and related to immunology [51]. MPO was also reported to be associated with another F3 generation vinclozolin lineage [37].

4.1.14 HAND2

The protein encoded by HAND2 belongs to the basic helix-loop–helix family of transcription factors and has critical roles in endometrial cancer [52].

4.1.15 CCL3

CCL3 is a cytokine belonging to the CC chemokine family that is involved in the recruitment and activation of polymorphonuclear leukocytes during acute inflammation. In experimental autoimmune orchitis, the highest content of CCL3 is in the testicular fluid and is associated with the onset of the disease [53]. In autoimmune orchitis, chronic testicular inflammation, chemokines such as CCL2, CCL3, and CCL4 attract immune cells within the testicular interstitium [54]. CCL3 levels were notably increased in human and mouse benign prostatic hyperplasia prostate glands [55]. Interleukin-6 (IL-6) trans-signaling via its soluble receptor sIL-6R governs the influx of innate immune cells to inflammatory foci through the regulation of the chemokine CCL3 [56]. The CCL3-CCR5 axis regulates the intratumoral accumulation of leukocytes and fibroblasts and promotes angiogenesis in murine lung metastasis [57].

4.1.16 HBE2

HBE2 does not have any orthologs to human genes and information regarding its functions is limited. HBE2 is a part of the hemoglobin complex that transfers oxygen in the blood. There have been no reported relationships between HBE2 and disease.

4.1.17 CMKLR1

CMKLR1, also known as CHEMR23, is a G protein-coupled receptor for the chemoattractant adipokine chemerin and the omega-3 fatty acid derived molecule resolvin E1. CMKLR1 is localized specifically in the Leydig cells of human and rat testes [58]. The CHEMR23/Chemerin axis may have a role in the recruitment of dendritic cells within the kidney in patients affected by lupus nephritis [59]. RvE1/ChEMR23-dependent rS6 phosphorylation occurs in macrophages [60]. CMKLR1 plays roles in the regulation of immune responses [61].

4.1.18 DBH

Dopamine beta-hydroxylase (DBH) mediates dopamine related reactions, is related to kidney function and its role is inheritable [62]. DBH is expressed in the testes [63] and in prostate cancer [64]. DBH has a relationship with immunology [65].

4.1.19 KCNT1

KCNT1 is a member of the calcium-activated potassium channel protein family that is important for kidney functions [66].

4.1.20 TAAR7B

TAAR7B is a member of the trace amine-associated receptors, but does not have a human ortholog, while TAAR1, a member of the human trace amine-associated receptors, has a relationship with the kidney [67].

4.1.21 Fibrinogen beta chain

The fibrinogen beta chain is a blood-borne glycoprotein composed of three pairs of nonidentical polypeptide chains. Heterozygosity for fibrinogen results in the efficient resolution of kidney ischemia reperfusion injury [68]. The association of elevated plasma fibrinogen levels with cancer-specific and overall survival was observed in prostate cancer patients [69]. Immune complexes containing citrullinated fibrinogen co-stimulated macrophages via Toll-like receptor 4 and Fcγ receptor [70].

4.1.22 BMP3

BMP3 is a member of the transforming growth factor beta superfamily. Developing human lung and kidney are major sites for the synthesis of BMP3 [71]. BMP3 mRNA was expressed predominantly in the rat prostate adenocarcinoma, PAIII [72].

4.1.23 ACTG2

ACTG2 encodes actin, a gamma-enteric smooth muscle. ACTG2 was overexpressed in a model of recessive polycystic kidney disease [73].

4.1.24 AQP2

AQP2 is found in the apical cell membranes of kidney collecting duct principal cells and in intracellular vesicles located throughout the cell. AQP2 is expressed in the kidney [74]. AQP2 was localized to Leydig cells, elongated spermatids and round spermatids [75]. Genetic polymorphisms in AQP2 might contribute to chemotherapy responses in lung cancer patients [76].

4.2 Genes identified by limma based FE

4.2.1 qk

MicroRNA-155 promotes the proliferation and invasion abilities of colon cancer cells by targeting quaking (qk) [77]. The tumor suppressing effects of QKI-5 are encoded by the qk gene in prostate cancer [78]. Lipid and fatty acid composition of testes of quaking mice was previously measured [79].

4.2.2 TOP1

Correlation between TOP1 and tyrosyl-DNA phosphodiesterase 1 activities in non-small-cell lung cancer tissues were reported [80]. A novel small molecule hybrid of vorinostat and a topoisomerase inhibitor displays anticancer activity against human hormone-refractory metastatic prostate cancer through dual inhibition of histone deacetylase and TOP1 [81].

4.2.3 Arhgef1

Arhgef1 is known to play roles in cancer [82]. Arhgef1 regulates alpha5beta1 integrin-mediated matrix metalloproteinase expression and is required for homeostatic lung immunity [83].

4.2.4 TEAD2

TEAD2, a Hippo pathway gene, is somatically mutated in gastric and colorectal cancers with high microsatellite instability [84].

4.2.5 Sirt2

A progressive increase in the expression of both SIRT2 and SIRT7 was noted during cancer progression [85]. SIRT2 expression was lower in human prostate cancer [86]. SIRT2 regulates lipopolysaccharide-induced renal tubular CXCL2 and CCL2 expression in kidneys [87].

4.2.6 gmfg

High GMFG expression correlates with poor prognosis and promotes cell migration and invasion in epithelial ovarian cancer [88].

4.2.7 alkbh6

ALKBH6 genes may play important roles in embryonal rhabdomyosarcoma [89].

4.2.8 MCEE

MCEE mutation was observed in gastric and colorectal cancers [90].

4.2.9 hbs1l

Genetic variation at HBS1L-MYB predisposes to myeloproliferative neoplasms [91].

4.2.10 HSPBP1

HSPBP1 expression was reported in leukemia [92].

4.2.11 XRCC1

Polymorphisms of XRCC1 [93] is related to prostate cancer. Transcription of the XRCC1 gene in kidneys of radiosensitive and radioresistant mice following whole-body irradiation [94]. XRCC1 expression is radiobiologically activated in sertoli cells in testis [95].

4.3 Genes identified by SAM based FE, but not by PCA based unsupervised FE, when $N' = 1000$

4.3.1 MYL1

The methylation status of histone H3 lysine 27 in the promoter region of MYL1 was altered by JARID2, which is a direct target of the PAX3-FOXO1 fusion protein, and inhibits myogenic differentiation of rhabdomyosarcoma cells [96].

4.3.2 SLC28A1

SLC28A1 was reported to be downregulated in neoplastic pancreatic tissues [97]. SLC28A1 was expressed in rat glomerulus [98].

4.3.3 PGAM2

Inhibition of PGAM2 by small RNAi or small molecule attenuated tumor growth [99]. PGAM2 was expressed in testis [100].

4.3.4 ALB

Binding of serum albumin on tumor cells was observed [101].

4.3.5 SLC13A3

SLC13A3 plays some roles in prostate cancer [102]. SLC13A3 was expressed in kidneys [103].

4.3.6 TTR

TTR is biomarker of pancreatic ductal adenocarcinoma [104] and prostate cancer [105]. Transthyretin amyloidosis is related to kidney [106]. TTR was observed in fish testis [107]. TTR is functionally related to immunology [108].

4.3.7 ANGPTL1

ANGPTL1 suppresses cancer cell motility [109].

4.3.8 TUBB3

TUBB3 was expressed in non-small-cell lung cancer [110]. TUBB3 was upregulated in prostate cancer [111] and in kidney cancer [112]. TUBB3 is expressed in testis [113].

4.3.9 IL15

IL-15 is a famous cancer therapy target [114]. IL-15 has critical roles in prostate cancer [115]. IL-15 plays critical roles in kidney cancer [116]. An IL-15 isoform is expressed in testis [117]. IL-15 dendritic cells are vaccine candidates for cancer immunotherapy [118].

4.3.10 BACH1

BACH1 plays critical roles in renal cancer [118] and prostate cancer [119].

4.3.11 ZFP36L2

Deletion of ZFP36L2 is related to tumor progression [120]. Functional regulation of ZFP36L2 occurs in response to lipopolysaccharide in mouse RAW264.7 macrophages [121].

References

[1] Singh, R., Sobhia, M.E.: Structure prediction and molecular dynamics simulations of a G-protein coupled receptor: human CCR2 receptor. J. Biomol. Struct. Dyn. **31**(7), 694–715 (2013)

[2] Kitagawa, K., Wada, T., Furuichi, K., Hashimoto, H., Ishiwata, Y., Asano, M., Takeya, M., Kuziel, W.A., Matsushima, K., Mukaida, N., Yokoyama, H.: Blockade of CCR2 ameliorates progressive fibrosis in kidney. Am. J. Pathol. **165**(1), 237–246 (2004)

[3] Ding, X., Yang, D.R., Lee, S.O., Chen, Y.L., Xia, L., Lin, S.J., Yu, S., Niu, Y.J., Li, G., Chang, C.: TR4 nuclear receptor promotes prostate cancer metastasis via upregulation of CCL2/CCR2 signaling. Int. J. Cancer **136**(4), 955–964 (2015)

[4] Lanca, T., Costa, M.F., Goncalves-Sousa, N., Rei, M., Grosso, A.R., Penido, C., Silva-Santos, B.: Protective role of the inflammatory CCR2/CCL2 chemokine pathway through recruitment of type 1 cytotoxic $\gamma\sigma$ T lymphocytes to tumor beds. J. Immunol. **190**(12), 6673–6680 (2013)

[5] Kobe, B., Kajava, A.V.: The leucine-rich repeat as a protein recognition motif. Curr. Opin. Struct. Biol. **11**(6), 725–732 (2001)

[6] Akter, J., Takatori, A., Hossain, M.S., Ozaki, T., Nakazawa, A., Ohira, M., Suenaga, Y., Nakagawara, A.: Expression of NLRR3 orphan receptor gene is negatively regulated by MYCN and Miz-1, and its downregulation is associated with unfavorable outcome in neuroblastoma. Clin. Cancer Res. **17**(21), 6681–6692 (2011)

[7] Chou, J.P., Ramirez, C.M., Wu, J.E., Effros, R.B.: Accelerated aging in HIV/AIDS: novel biomarkers of senescent human CD8+ T cells. PLoS ONE **8**(5), 64702 (2013)

[8] Beischlag, T.V., Luis Morales, J., Hollingshead, B.D., Perdew, G.H.: The aryl hydrocarbon receptor complex and the control of gene expression. Crit. Rev. Eukaryot. Gene Expr. **18**(3), 207–250 (2008)

[9] Nguyen, L.P., Bradfield, C.A.: The search for endogenous activators of the aryl hydrocarbon receptor. Chem. Res. Toxicol. **21**(1), 102–116 (2008)

[10] Barisione, C., Ghigliotti, G., Canepa, M., Balbi, M., Brunelli, C., Ameri, P.: Indoxyl sulfate: a candidate target for the prevention and treatment of cardiovascular disease in chronic kidney disease. Curr Drug Targets **16**(4), 366–372 (2015)

[11] Hansen, D.A., Esakky, P., Drury, A., Lamb, L., Moley, K.H.: The aryl hydrocarbon receptor is important for proper seminiferous tubule architecture and sperm development in mice. Biol. Reprod. **90**(1), 8 (2014)

[12] Palkova, L., Vondra?ek, J., Trilecova, L., Ciganek, M., P?n?ikova, K., Ne?a, J., Milcova, A., Topinka, J., Machala, M.: The aryl hydrocarbon receptor-mediated and genotoxic effects of fractionated extract of standard reference diesel exhaust particle material in pulmonary, liver and prostate cells. Toxicol In Vitro **29**(3), 438–448 (2014)

[13] Umeyama, H., Iwadate, M., Taguchi, Y.H.: TINAGL1 and B3GALNT1 are potential therapy target genes to suppress metastasis in non-small cell lung cancer. BMC Genomics **15 Suppl 9**, 2 (2014)

[14] Esser, C., Rannug, A.: The Aryl Hydrocarbon Receptor in Barrier Organ Physiology, Immunology, and Toxicology. Pharmacol. Rev. **67**(2), 259–279 (2015)

[15] Beerlage, C., Greb, J., Kretschmer, D., Assaggaf, M., Trackman, P.C., Hansmann, M.L., Bonin, M., Eble, J.A., Peschel, A., Brune, B., Kempf, V.A.: Hypoxia-inducible factor 1-regulated lysyl oxidase is involved in Staphylococcus aureus abscess formation. Infect. Immun. **81**(7), 2562–2573 (2013)

[16] Bais, M.V., Ozdener, G.B., Sonenshein, G.E., Trackman, P.C.: Effects of tumor-suppressor lysyl oxidase propeptide on prostate cancer xenograft growth and its direct interactions with DNA repair pathways. Oncogene **34**(15), 1928–1937 (2015)

[17] Wood, H.M., Lee, U.J., Vurbic, D., Sabanegh, E., Ross, J.H., Li, T., Damaser, M.S.: Sexual development and fertility of Loxl1-/- male mice. J. Androl. **30**(4), 452–459 (2009)

[18] Cox, T.R., Bird, D., Baker, A.M., Barker, H.E., Ho, M.W., Lang, G., Erler, J.T.: LOX-mediated collagen crosslinking is responsible for fibrosis-enhanced metastasis. Cancer Res. **73**(6), 1721–1732 (2013)

[19] El-Haibi, C.P., Bell, G.W., Zhang, J., Collmann, A.Y., Wood, D., Scherber, C.M., Csizmadia, E., Mariani, O., Zhu, C., Campagne, A., Toner, M., Bhatia, S.N., Irimia, D., Vincent-Salomon, A., Karnoub, A.E.: Critical role for lysyl oxidase in mesenchymal stem cell-driven breast cancer malignancy. Proc. Natl. Acad. Sci. U.S.A. **109**(43), 17460–17465 (2012)

[20] Nishioka, T., Eustace, A., West, C.: Lysyl oxidase: from basic science to future cancer treatment. Cell Struct. Funct. **37**(1), 75–80 (2012)

[21] Wadelin, F., Fulton, J., McEwan, P.A., Spriggs, K.A., Emsley, J., Heery, D.M.: Leucine-rich repeat protein PRAME: expression, potential functions and clinical implications for leukaemia. Mol. Cancer **9**, 226 (2010)

[22] Mistry, B.V., Zhao, Y., Chang, T.C., Yasue, H., Chiba, M., Oatley, J., Diaz, F., Liu, W.S.: Differential expression of PRAMEL1, a cancer/testis antigen, during spermatogenesis in the mouse. PLoS ONE **8**(4), 60611 (2013)

[23] Kinney, S.R., Moser, M.T., Pascual, M., Greally, J.M., Foster, B.A., Karpf, A.R.: Opposing roles of Dnmt1 in early- and late-stage murine prostate cancer. Mol. Cell. Biol. **30**(17), 4159–4174 (2010)

[24] Yunta, M., Rodriguez-Barbero, A., Arevalo, M.A., Lopez-Novoa, J.M., Lazo, P.A.: Induction of DNA synthesis by ligation of the CD53 tetraspanin antigen in primary cultures of mesangial cells. Kidney Int. **63**(2), 534–542 (2003)

[25] Todros-Dawda, I., Kveberg, L., Vaage, J.T., Inngjerdingen, M.: The tetraspanin CD53 modulates responses from activating NK cell receptors, promoting LFA-1 activation and dampening NK cell effector functions. PLoS ONE **9**(5), 97844 (2014)

[26] Li, K.J., Wu, C.H., Hsieh, S.C., Lu, M.C., Tsai, C.Y., Yu, C.L.: Deranged bioenergetics and defective redox capacity in T lymphocytes and neutrophils are related to cellular dysfunction and increased oxidative stress in patients with active systemic lupus erythematosus. Clin. Dev. Immunol. **2012**, 548516 (2012)

[27] Mansson, R., Lagergren, A., Hansson, F., Smith, E., Sigvardsson, M.: The CD53 and CEACAM-1 genes are genetic targets for early B cell factor. Eur. J. Immunol. **37**(5), 1365–1376 (2007)

[28] Ross, R.W., Galsky, M.D., Scher, H.I., Magidson, J., Wassmann, K., Lee, G.S., Katz, L., Subudhi, S.K., Anand, A., Fleisher, M., Kantoff, P.W., Oh, W.K.: A whole-blood RNA transcript-based prognostic model in men with castration-resistant prostate cancer: a prospective study. Lancet Oncol. **13**(11), 1105–1113 (2012)

[29] Rabb, H., Mendiola, C.C., Dietz, J., Saba, S.R., Issekutz, T.B., Abanilla, F., Bonventre, J.V., Ramirez, G.: Role of CD11a and CD11b in ischemic acute renal failure in rats. Am. J. Physiol. **267**(6 Pt 2), 1052–1058 (1994)

[30] Horstmann, W.G., Timens, W.: Lack of adhesion molecules in testicular diffuse centroblastic and immunoblastic B cell lymphomas as a contributory factor in malignant behaviour. Virchows Arch. **429**(2-3), 83–90 (1996)

[31] Wang, Y., Shu, Y., Xiao, Y., Wang, Q., Kanekura, T., Li, Y., Wang, J., Zhao, M., Lu, Q., Xiao, R.: Hypomethylation and overexpression of ITGAL (CD11a) in CD4(+) T cells in systemic sclerosis. Clin Epigenetics **6**(1), 25 (2014)

[32] Skinner, M.K., Anway, M.D., Savenkova, M.I., Gore, A.C., Crews, D.: Transgenerational epigenetic programming of the brain transcriptome and anxiety behavior. PLoS ONE **3**(11), 3745 (2008)

[33] Sugimura, K., Tanaka, T., Tanaka, Y., Takano, H., Kanagawa, K., Sakamoto, N., Ikemoto, S., Kawashima, H., Nakatani, T.: Decreased sulfotransferase SULT1C2 gene expression in DPT-induced polycystic kidney. Kidney Int. **62**(3), 757–762 (2002)

[34] Zhou, X.J., Cheng, F.J., Qi, Y.Y., Zhao, Y.F., Hou, P., Zhu, L., Lv, J.C., Zhang, H.: FCGR2B and FCRLB gene polymorphisms associated with IgA nephropathy. PLoS ONE **8**(4), 61208 (2013)

[35] Sun, J.B., Xiang, Z., Smith, K.G., Holmgren, J.: Important role for FcγRIIB on B lymphocytes for mucosal antigen-induced tolerance and Foxp3+ regulatory T cells. J. Immunol. **191**(8), 4412–4422 (2013)

[36] Zadravec, D., Tvrdik, P., Guillou, H., Haslam, R., Kobayashi, T., Napier, J.A., Capecchi, M.R., Jacobsson, A.: ELOVL2 controls the level of n-6 28:5 and 30:5 fatty acids in testis, a prerequisite for male fertility and sperm maturation in mice. J. Lipid Res. **52**(2), 245–255 (2011)

[37] Skinner, M.K., Savenkova, M.I., Zhang, B., Gore, A.C., Crews, D.: Gene bionetworks involved in the epigenetic transgenerational inheritance of altered mate preference: environmental epigenetics and evolutionary biology. BMC Genomics **15**, 377 (2014)

[38] Zhang, L., Zhu, Y., Zhang, D., Zhang, J., Tian, Y.: Platelet factor 4 protects kidney allograft in a rat kidney transplantation model. Inflammation **38**(2), 520–526 (2015)

[39] Magre, S., Rebourcet, D., Ishaq, M., Wargnier, R., Debard, C., Meugnier, E., Vidal, H., Cohen-Tannoudji, J., Le Magueresse-Battistoni, B.: Gender differences in transcriptional signature of developing rat testes and ovaries following embryonic exposure to 2,3,7,8-TCDD. PLoS ONE **7**(7), 40306 (2012)

[40] Cervi, D., Pak, B., Venier, N.A., Sugar, L.M., Nam, R.K., Fleshner, N.E., Klotz, L.H., Venkateswaran, V.: Micronutrients attenuate progression of prostate cancer by elevating the endogenous inhibitor of angiogenesis, platelet factor-4. BMC Cancer **10**, 258 (2010)

[41] Schwartzkopff, F., Petersen, F., Grimm, T.A., Brandt, E.: CXC chemokine ligand 4 (CXCL4) down-regulates CC chemokine receptor expression on human monocytes. Innate Immun **18**(1), 124–139 (2012)

[42] Rivera, I., Pinheiro, A., Silva, M.J., de Almeida, I.T.: The human testis-specific PDHA2 gene: Functional role, regulatory mechanisms and potential therapeutic target. In: iConcept Press Ltd. (ed.) Endocrine Diseases, p. . iConcept Press Ltd., ??? (2015)

[43] Pinheiro, A., Faustino, I., Silva, M.J., Silva, J., Sa, R., Sousa, M., Barros, A., de Almeida, I.T., Rivera, I.: Human testis-specific PDHA2 gene: methylation status of a CpG island in the open reading frame correlates with transcriptional activity. Mol. Genet. Metab. **99**(4), 425–430 (2010)

[44] Pinheiro, A., Silva, M.J., Graca, I., Silva, J., Sa, R., Sousa, M., Barros, A., Tavares de Almeida, I., Rivera, I.: Pyruvate dehydrogenase complex: mRNA and protein expression patterns of E1α subunit genes in human spermatogenesis. Gene **506**(1), 173–178 (2012)

[45] Iannello, R.C., Young, J., Sumarsono, S., Tymms, M.J., Dahl, H.H., Gould, J., Hedger, M., Kola, I.: Regulation of Pdha-2 expression is mediated by proximal promoter sequences and CpG methylation. Mol. Cell. Biol. **17**(2), 612–619 (1997)

[46] Malle, E., Buch, T., Grone, H.J.: Myeloperoxidase in kidney disease. Kidney Int. **64**(6), 1956–1967 (2003)

[47] Madhusudhana Rao, A., Anand, U., Anand, C.V.: Myeloperoxidase in chronic kidney disease. Indian J Clin Biochem **26**(1), 28–31 (2011)

[48] Ozturk, H., Ozturk, H., Gideroglu, K., Terzi, H., Bugdayci, G.: Montelukast protects against testes ischemia/reperfusion injury in rats. Can Urol Assoc J **4**(3), 174–179 (2010)

[49] Roumeguere, T., Delree, P., Van Antwerpen, P., Rorive, S., Vanhamme, L., de Ryhove, L.d.e.L., Serteyn, D., Wespes, E., Vanhaerverbeek, M., Boudjeltia, K.Z.: Intriguing location of myeloperoxidase in the prostate: a preliminary immunohistochemical study. Prostate **72**(5), 507–513 (2012)

[50] Ding, G., Liu, F., Feng, C., Xu, J., Ding, Q.: [Association between the myeloperoxidase gene polymorphisms and the susceptibility to prostate cancer: a case-control study in a Chinese population]. Actas Urol Esp **37**(2), 79–82 (2013)

[51] Arnhold, J., Flemmig, J.: Human myeloperoxidase in innate and acquired immunity. Arch. Biochem. Biophys. **500**(1), 92–106 (2010)

[52] Jones, A., Teschendorff, A.E., Li, Q., Hayward, J.D., Kannan, A., Mould, T., West, J., Zikan, M., Cibula, D., Fiegl, H., Lee, S.H., Wik, E., Hadwin, R., Arora, R., Lemech, C., Turunen, H., Pakarinen, P., Jacobs, I.J., Salvesen, H.B., Bagchi, M.K., Bagchi, I.C., Widschwendter, M.: Role of DNA methylation and epigenetic silencing of HAND2 in endometrial cancer development. PLoS Med. **10**(11), 1001551 (2013)

[53] Guazzone, V.A., Jacobo, P., Denduchis, B., Lustig, L.: Expression of cell adhesion molecules, chemokines and chemokine receptors involved in leukocyte traffic in rats undergoing autoimmune orchitis. Reproduction **143**(5), 651–662 (2012)

[54] Guazzone, V.A., Jacobo, P., Theas, M.S., Lustig, L.: Cytokines and chemokines in testicular inflammation: A brief review. Microsc. Res. Tech. **72**(8), 620–628 (2009)

[55] Wang, X., Lin, W.J., Izumi, K., Jiang, Q., Lai, K.P., Xu, D., Fang, L.Y., Lu, T., Li, L., Xia, S., Chang, C.: Increased infiltrated macrophages in benign prostatic hyperplasia (BPH): role of stromal androgen receptor in macrophage-induced prostate stromal cell proliferation. J. Biol. Chem. **287**(22), 18376–18385 (2012)

[56] Ravi, A.K., Khurana, S., Lemon, J., Plumb, J., Booth, G., Healy, L., Catley, M., Vestbo, J., Singh, D.: Increased levels of soluble interleukin-6 receptor and CCL3 in COPD sputum. Respir. Res. **15**, 103 (2014)

[57] Wu, Y., Li, Y.Y., Matsushima, K., Baba, T., Mukaida, N.: CCL3-CCR5 axis regulates intratumoral accumulation of leukocytes and fibroblasts and promotes angiogenesis in murine lung metastasis process. J. Immunol. **181**(9), 6384–6393 (2008)

[58] Li, L., Ma, P., Huang, C., Liu, Y., Zhang, Y., Gao, C., Xiao, T., Ren, P.G., Zabel, B.A., Zhang, J.V.: Expression of chemerin and its receptors in rat testes and its action on testosterone secretion. J. Endocrinol. **220**(2), 155–163 (2014)

[59] De Palma, G., Castellano, G., Del Prete, A., Sozzani, S., Fiore, N., Loverre, A., Parmentier, M., Gesualdo, L., Grandaliano, G., Schena, F.P.: The possible role of ChemR23/Chemerin axis in the recruitment of dendritic cells in lupus nephritis. Kidney Int. **79**(11), 1228–1235 (2011)

[60] Fredman, G., Serhan, C.N.: Specialized proresolving mediator targets for RvE1 and RvD1 in peripheral blood and mechanisms of resolution. Biochem. J. **437**(2), 185–197 (2011)

[61] Zabel, B.A., Silverio, A.M., Butcher, E.C.: Chemokine-like receptor 1 expression and chemerin-directed chemotaxis distinguish plasmacytoid from myeloid dendritic cells in human blood. J. Immunol. **174**(1), 244–251 (2005)

[62] Pasha, D.N., Davis, J.T., Rao, F., Chen, Y., Wen, G., Fung, M.M., Mahata, M., Zhang, K., Trzebinska, D., Mustapic, M., Hightower, C.M., Lipkowitz, M.S., Ji, M., Ziegler, M.G., Nievergelt, C.M., O'Connor, D.T.: Heritable influence of DBH on adrenergic and renal function: twin and disease studies. PLoS ONE **8**(12), 82956 (2013)

[63] Gong, Y.G., Feng, M.M., Hu, X.N., Wang, Y.Q., Gu, M., Zhang, W., Ge, R.S.: Peptidergic not monoaminergic fibers profusely innervate the young adult human testis. J. Anat. **214**(3), 330–338 (2009)

[64] Crowe, R., Chapple, C.R., Burnstock, G.: The human prostate gland: a histochemical and immunohistochemical study of neuropeptides, serotonin, dopamine beta-hydroxylase and acetylcholinesterase in autonomic nerves and ganglia. Br J Urol **68**(1), 53–61 (1991)

[65] Alaniz, R.C., Thomas, S.A., Perez-Melgosa, M., Mueller, K., Farr, A.G., Palmiter, R.D., Wilson, C.B.: Dopamine beta-hydroxylase deficiency impairs cellular immunity. Proc. Natl. Acad. Sci. U.S.A. **96**(5), 2274–2278 (1999)

[66] Paulais, M., Lachheb, S., Teulon, J.: A Na+- and Cl- -activated K+ channel in the thick ascending limb of mouse kidney. J. Gen. Physiol. **127**(2), 205–215 (2006)

[67] Xie, Z., Westmoreland, S.V., Miller, G.M.: Modulation of monoamine transporters by common biogenic amines via trace amine-associated receptor 1 and monoamine autoreceptors in human embryonic kidney 293 cells and brain synaptosomes. J. Pharmacol. Exp. Ther. **325**(2), 629–640 (2008)

[68] Ajay, A.K., Saikumar, J., Bijol, V., Vaidya, V.S.: Heterozygosity for fibrinogen results in efficient resolution of kidney ischemia reperfusion injury. PLoS ONE **7**(9), 45628 (2012)

[69] Thurner, E.M., Krenn-Pilko, S., Langsenlehner, U., Stojakovic, T., Pichler, M., Gerger, A., Kapp, K.S., Langsenlehner, T.: The association of an elevated plasma fibrinogen level with cancer-specific and overall survival in prostate cancer patients. World J Urol (2014)

[70] Sokolove, J., Zhao, X., Chandra, P.E., Robinson, W.H.: Immune complexes containing citrullinated fibrinogen costimulate macrophages via Toll-like receptor 4 and Fcγ receptor. Arthritis Rheum. **63**(1), 53–62 (2011)

[71] Vukicevic, S., Helder, M.N., Luyten, F.P.: Developing human lung and kidney are major sites for synthesis of bone morphogenetic protein-3 (osteogenin). J. Histochem. Cytochem. **42**(7), 869–875 (1994)

[72] Harris, S.E., Harris, M.A., Mahy, P., Wozney, J., Feng, J.Q., Mundy, G.R.: Expression of bone morphogenetic protein messenger RNAs by normal rat and human prostate and prostate cancer cells. Prostate **24**(4), 204–211 (1994)

[73] Mrug, M., Zhou, J., Woo, Y., Cui, X., Szalai, A.J., Novak, J., Churchill, G.A., Guay-Woodford, L.M.: Overexpression of innate immune response genes in a model of recessive polycystic kidney disease. Kidney Int. **73**(1), 63–76 (2008)

[74] Takata, K., Matsuzaki, T., Tajika, Y., Ablimit, A., Hasegawa, T.: Localization and trafficking of aquaporin 2 in the kidney. Histochem. Cell Biol. **130**(2), 197–209 (2008)

[75] Klein, C., Troedsson, M.H., Rutllant, J.: Region-specific expression of aquaporin subtypes in equine testis, epididymis, and ductus deferens. Anat Rec (Hoboken) **296**(7), 1115–1126 (2013)

[76] Wang, Y., Yin, J.Y., Li, X.P., Chen, J., Qian, C.Y., Zheng, Y., Fu, Y.L., Chen, Z.Y., Zhou, H.H., Liu, Z.Q.: The association of transporter genes polymorphisms and lung cancer chemotherapy response. PLoS ONE **9**(3), 91967 (2014)

[77] He, B., Gao, S.Q., Huang, L.D., Huang, Y.H., Zhang, Q.Y., Zhou, M.T., Shi, H.Q., Song, Q.T., Shan, Y.F.: MicroRNA-155 promotes the proliferation and invasion abilities of colon cancer cells by targeting quaking. Mol Med Rep **11**(3), 2355–2359 (2015)

[78] Zhao, Y., Zhang, G., Wei, M., Lu, X., Fu, H., Feng, F., Wang, S., Lu, W., Wu, N., Lu, Z., Yuan, J.: The tumor suppressing effects of QKI-5 in prostate cancer: a novel diagnostic and prognostic protein. Cancer Biol. Ther. **15**(1), 108–118 (2014)

[79] Coniglio, J.G., Grogan, W.M., Harris, D.G., Fitzhugh, M.L.: Lipid and fatty acid composition of testes of quaking mice. Lipids **10**(2), 109–112 (1975)

[80] Jakobsen, A.K., Lauridsen, K.L., Samuel, E.B., Proszek, J., Knudsen, B.R., Hager, H., Stougaard, M.: Correlation between topoisomerase I and tyrosyl-DNA phosphodiesterase 1 activities in non-small cell lung cancer tissue. Exp. Mol. Pathol. **99**(1), 56–64 (2015)

[81] Yu, C.C., Pan, S.L., Chao, S.W., Liu, S.P., Hsu, J.L., Yang, Y.C., Li, T.K., Huang, W.J., Guh, J.H.: A novel small molecule hybrid of vorinostat and DACA displays anticancer activity against human hormone-refractory metastatic prostate cancer through dual inhibition of histone deacetylase and topoisomerase I. Biochem. Pharmacol. **90**(3), 320–330 (2014)

[82] Yu, O.M., Brown, J.H.: G Protein-Coupled Receptor and RhoA-Stimulated Transcriptional Responses: Links to Inflammation, Differentiation, and Cell Proliferation. Mol. Pharmacol. **88**(1), 171–180 (2015)

[83] Hartney, J.M., Brown, J., Chu, H.W., Chang, L.Y., Pelanda, R., Torres, R.M.: Arhgef1 regulates alpha5beta1 integrin-mediated matrix metalloproteinase expression and is required for homeostatic lung immunity. Am. J. Pathol. **176**(3), 1157–1168 (2010)

[84] Je, E.M., Choi, Y.J., Chung, Y.J., Yoo, N.J., Lee, S.H.: TEAD2, a Hippo pathway gene, is somatically mutated in gastric and colorectal cancers with high microsatellite instability. APMIS **123**(4), 359–360 (2015)

[85] Singh, S., Kumar, P.U., Thakur, S., Kiran, S., Sen, B., Sharma, S., Rao, V.V., Poongothai, A.R., Ramakrishna, G.: Expression/localization patterns of sirtuins (SIRT1, SIRT2, and SIRT7) during progression of cervical cancer and effects of sirtuin inhibitors on growth of cervical cancer cells. Tumour Biol. (2015)

[86] Hou, H., Chen, W., Zhao, L., Zuo, Q., Zhang, G., Zhang, X., Wang, H., Gong, H., Li, X., Wang, M., Wang, Y., Li, X.: Cortactin is associated with tumour progression and poor prognosis in prostate cancer and SIRT2 other than HADC6 may work as facilitator in situ. J. Clin. Pathol. **65**(12), 1088–1096 (2012)

[87] Jung, Y.J., Lee, A.S., Nguyen-Thanh, T., Kim, D., Kang, K.P., Lee, S., Park, S.K., Kim, W.: SIRT2 Regulates LPS-Induced Renal Tubular CXCL2 and CCL2 Expression. J. Am. Soc. Nephrol. **26**(7), 1549–1560 (2015)

[88] Zuo, P., Ma, Y., Huang, Y., Ye, F., Wang, P., Wang, X., Zhou, C., Lu, W., Kong, B., Xie, X.: High GMFG expression correlates with poor prognosis and promotes cell migration and invasion in epithelial ovarian cancer. Gynecol. Oncol. **132**(3), 745–751 (2014)

[89] Liu, C., Li, D., Hu, J., Jiang, J., Zhang, W., Chen, Y., Cui, X., Qi, Y., Zou, H., Zhang, W., Li, F.: Chromosomal and genetic imbalances in Chinese patients with rhabdomyosarcoma detected by high-resolution array comparative genomic hybridization. Int J Clin Exp Pathol **7**(2), 690–698 (2014)

[90] Oh, H.R., An, C.H., Yoo, N.J., Lee, S.H.: Somatic mutations of amino acid metabolism-related genes in gastric and colorectal cancers and their regional heterogeneity–a short report. Cell Oncol (Dordr) **37**(6), 455–461 (2014)

[91] Tapper, W., Jones, A.V., Kralovics, R., Harutyunyan, A.S., Zoi, K., Leung, W., Godfrey, A.L., Guglielmelli, P., Callaway, A., Ward, D., Aranaz, P., White, H.E., Waghorn, K., Lin, F., Chase, A., Baxter, E.J., Maclean, C., Nangalia, J., Chen, E., Evans, P., Short, M., Jack, A., Wallis, L., Oscier, D., Duncombe, A.S., Schuh, A., Mead, A.J., Griffiths, M., Ewing, J., Gale, R.E., Schnittger, S., Haferlach, T., Stegelmann, F., Dohner, K., Grallert, H., Strauch, K., Tanaka, T., Bandinelli, S., Giannopoulos, A., Pieri, L., Mannarelli, C., Gisslinger, H., Barosi, G., Cazzola, M., Reiter, A., Harrison, C., Campbell, P., Green, A.R., Vannucchi, A., Cross, N.C.: Genetic variation at MECOM, TERT, JAK2 and HBS1L-MYB predisposes to myeloproliferative neoplasms. Nat Commun **6**, 6691 (2015)

[92] Sedlackova, L., Spacek, M., Holler, E., Imryskova, Z., Hromadnikova, I.: Heat-shock protein expression in leukemia. Tumour Biol. **32**(1), 33–44 (2011)

[93] Zhu, H., Jiu, T., Wang, D.: Impact of polymorphisms of the DNA repair gene XRCC1 and their role in the risk of prostate cancer. Pak J Med Sci **31**(2), 290–294 (2015)

[94] Labudova, O., Hardmeier, R., Kitzmuller, E., Rink, H., Lubec, G.: Transcription of the XRCC1 gene in kidneys of radiosensitive and radioresistant mice following whole-body irradiation. Nephron **79**(1), 61–66 (1998)

[95] Ahmed, E.A., Barten-van Rijbroek, A.D., Kal, H.B., Sadri-Ardekani, H., Mizrak, S.C., van Pelt, A.M., de Rooij, D.G.: Proliferative activity in vitro and DNA repair indicate that adult mouse and human Sertoli cells are not terminally differentiated, quiescent cells. Biol. Reprod. **80**(6), 1084–1091 (2009)

[96] Walters, Z.S., Villarejo-Balcells, B., Olmos, D., Buist, T.W., Missiaglia, E., Allen, R., Al-Lazikani, B., Garrett, M.D., Blagg, J., Shipley, J.: JARID2 is a direct target of the PAX3-FOXO1 fusion protein and inhibits myogenic differentiation of rhabdomyosarcoma cells. Oncogene **33**(9), 1148–1157 (2014)

[97] Mohelnikova-Duchonova, B., Brynychova, V., Hlavac, V., Kocik, M., Oliverius, M., Hlavsa, J., Honsova, E., Mazanec, J., Kala, Z., Melichar, B., Soucek, P.: The association between the expression of solute carrier transporters and the prognosis of pancreatic cancer. Cancer Chemother. Pharmacol. **72**(3), 669–682 (2013)

[98] Rodriguez-Mulero, S., Errasti-Murugarren, E., Ballarin, J., Felipe, A., Doucet, A., Casado, F.J., Pastor-Anglada, M.: Expression of concentrative nucleoside transporters SLC28 (CNT1, CNT2, and CNT3) along the rat nephron: effect of diabetes. Kidney Int. **68**(2), 665–672 (2005)

[99] Conley, J.F., Martin, R.: Spotlight on AIDS, dental auxiliaries. J Calif Dent Assoc **16**(11), 61–63 (1988)

[100] Zhang, J., Yu, L., Fu, Q., Gao, J., Xie, Y., Chen, J., Zhang, P., Liu, Q., Zhao, S.: Mouse phosphoglycerate mutase M and B isozymes: cDNA cloning, enzyme activity assay and mapping. Gene **264**(2), 273–279 (2001)

[101] Wang, J., Ueno, H., Masuko, T., Hashimoto, Y.: Binding of serum albumin on tumor cells and characterization of the albumin binding protein. J. Biochem. **115**(5), 898–903 (1994)

[102] Zhunussova, A., Sen, B., Friedman, L., Tuleukhanov, S., Brooks, A.D., Sensenig, R., Orynbayeva, Z.: Tumor microenvironment promotes dicarboxylic acid carrier-mediated transport of succinate to fuel prostate cancer mitochondria. Am J Cancer Res **5**(5), 1665–1679 (2015)

[103] Schlessinger, A., Sun, N.N., Colas, C., Pajor, A.M.: Determinants of substrate and cation transport in the human Na+/dicarboxylate cotransporter NaDC3. J. Biol. Chem. **289**(24), 16998–17008 (2014)

[104] Chen, J., Chen, L.J., Xia, Y.L., Zhou, H.C., Yang, R.B., Wu, W., Lu, Y., Hu, L.W., Zhao, Y.: Identification and verification of transthyretin as a potential biomarker for pancreatic ductal adenocarcinoma. J. Cancer Res. Clin. Oncol. **139**(7), 1117–1127 (2013)

[105] Wang, D., Liang, H., Mao, X., Liu, W., Li, M., Qiu, S.: Changes of transthyretin and clusterin after androgen ablation therapy and correlation with prostate cancer malignancy. Transl Oncol **5**(2), 124–132 (2012)

[106] Lobato, L., Rocha, A.: Transthyretin amyloidosis and the kidney. Clin J Am Soc Nephrol **7**(8), 1337–1346 (2012)

[107] Santos, C.R., Anjos, L., Power, D.M.: Transthyretin in fish: state of the art. Clin. Chem. Lab. Med. **40**(12), 1244–1249 (2002)

[108] Planque, S.A., Nishiyama, Y., Hara, M., Sonoda, S., Murphy, S.K., Watanabe, K., Mitsuda, Y., Brown, E.L., Massey, R.J., Primmer, S.R., O'Nuallain, B., Paul, S.: Physiological IgM class catalytic antibodies selective for transthyretin amyloid. J. Biol. Chem. **289**(19), 13243–13258 (2014)

[109] Kuo, T.C., Tan, C.T., Chang, Y.W., Hong, C.C., Lee, W.J., Chen, M.W., Jeng, Y.M., Chiou, J., Yu, P., Chen, P.S., Wang, M.Y., Hsiao, M., Su, J.L., Kuo, M.L.: Angiopoietin-like protein 1 suppresses SLUG to inhibit cancer cell motility. J. Clin. Invest. **123**(3), 1082–1095 (2013)

[110] Qiao, H., Huang, X., Guo, H., Liu, Y., Yue, C.: ERCC1, RRM1 and TUBB3 mRNA expression on the tumor response and overall survival of non-small cell lung cancer treated with platinum-based chemotherapy. Pak J Med Sci **30**(6), 1403–1408 (2014)

[111] Nordin, A., Wang, W., Welen, K., Damber, J.E.: Midkine is associated with neuroendocrine differentiation in castration-resistant prostate cancer. Prostate **73**(6), 657–667 (2013)

[112] Quaas, A., Rahvar, A.H., Burdelski, C., Koop, C., Eichelberg, C., Rink, M., Dahlem, R., Schlomm, T., Tsourlakis, M.C., Simon, R., Minner, S., Sauter, G., Steurer, S.: III-tubulin overexpression is linked to aggressive tumor features and shortened survival in clear cell renal cell carcinoma. World J Urol (2014)

[113] De Gendt, K., Denolet, E., Willems, A., Daniels, V.W., Clinckemalie, L., Denayer, S., Wilkinson, M.F., Claessens, F., Swinnen, J.V., Verhoeven, G.: Expression of Tubb3, a beta-tubulin isotype, is regulated by androgens in mouse and rat Sertoli cells. Biol. Reprod. **85**(5), 934–945 (2011)

[114] Waldmann, T.A.: Interleukin-15 in the treatment of cancer. Expert Rev Clin Immunol **10**(12), 1689–1701 (2014)

[115] Morris, J.C., Ramlogan-Steel, C.A., Yu, P., Black, B.A., Mannan, P., Allison, J.P., Waldmann, T.A., Steel, J.C.: Vaccination with tumor cells expressing IL-15 and IL-15R ± inhibits murine breast and prostate cancer. Gene Ther. **21**(4), 393–401 (2014)

[116] Giron-Michel, J., Azzi, S., Khawam, K., Mortier, E., Caignard, A., Devocelle, A., Ferrini, S., Croce, M., Francois, H., Lecru, L., Charpentier, B., Chouaib, S., Azzarone, B., Eid, P.: Interleukin-15 plays a central role in human kidney physiology and cancer through the c signaling pathway. PLoS ONE **7**(2), 31624 (2012)

[117] Tagaya, Y., Kurys, G., Thies, T.A., Losi, J.M., Azimi, N., Hanover, J.A., Bamford, R.N., Waldmann, T.A.: Generation of secretable and nonsecretable interleukin 15 isoforms through alternate usage of signal peptides. Proc. Natl. Acad. Sci. U.S.A. **94**(26), 14444–14449 (1997)

[118] Anguille, S., Lion, E., Van den Bergh, J., Van Acker, H.H., Willemen, Y., Smits, E.L., Van Tendeloo, V.F., Berneman, Z.N.: Interleukin-15 dendritic cells as vaccine candidates for cancer immunotherapy. Hum Vaccin Immunother **9**(9), 1956–1961 (2013)

[119] Alvarez, A., Woolf, P.J.: RegNetB: predicting relevant regulator-gene relationships in localized prostate tumor samples. BMC Bioinformatics **12**, 243 (2011)

[120] Hodson, D.J., Janas, M.L., Galloway, A., Bell, S.E., Andrews, S., Li, C.M., Pannell, R., Siebel, C.W., MacDonald, H.R., De Keersmaecker, K., Ferrando, A.A., Grutz, G., Turner, M.: Deletion of the RNA-binding proteins ZFP36L1 and ZFP36L2 leads to perturbed thymic development and T lymphoblastic leukemia. Nat. Immunol. **11**(8), 717–724 (2010)

[121] Wang, K.T., Wang, H.H., Wu, Y.Y., Su, Y.L., Chiang, P.Y., Lin, N.Y., Wang, S.C., Chang, G.D., Chang, C.J.: Functional regulation of Zfp36l1 and Zfp36l2 in response to lipopolysaccharide in mouse RAW264.7 macrophages. J Inflamm (Lond) **12**, 42 (2015)
