# Supplementary material for: Identification of aberrant gene expression associated with aberrant promoter methylation in primordial germ cells between E13 and E16 rat F3 generation vinclozolin lineage
Source: BMC Bioinformatics. 2015 Dec 9;16(Suppl 18):S16. doi: 10.1186/1471-2105-16-S18-S16 (PMC4682393; doi:10.1186/1471-2105-16-S18-S16)
Supplement: Additional file 2 — List of genes selected by PCA-based unsupervised FE, limma-based FE, and SAM-based FE. [file 1471-2105-16-S18-S16-S2.pdf]

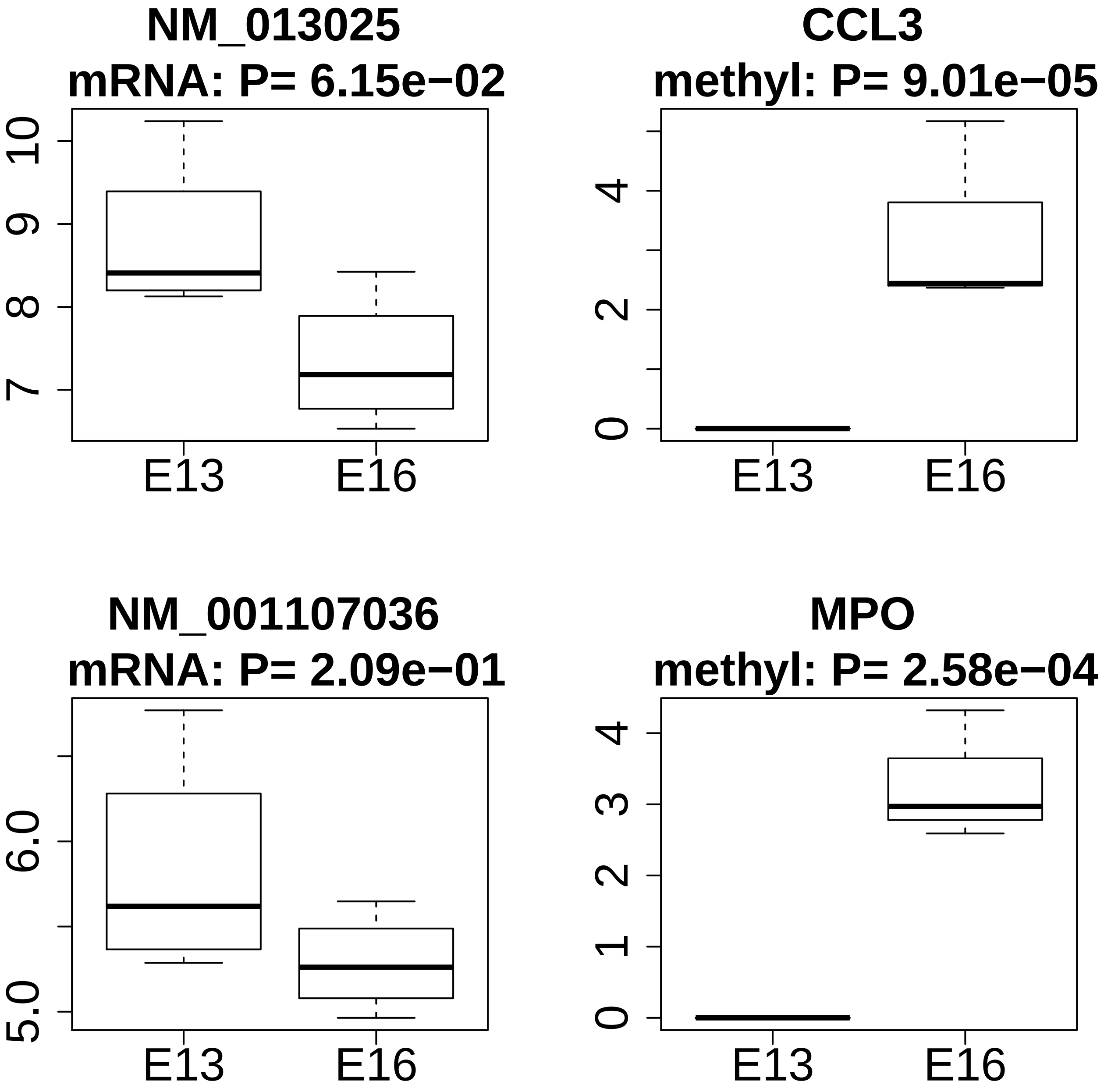
NM_013025
mRNA: P= 6.15e−02
10
9
8
7
E13
E16
CCL3
methyl: P= 9.01e−05
4
2
0
E13
E16
NM_001107036
mRNA: P= 2.09e−01
6.0
5.0
E13
E16
MPO
methyl: P= 2.58e−04
4
3
2
1
0
E13
E16

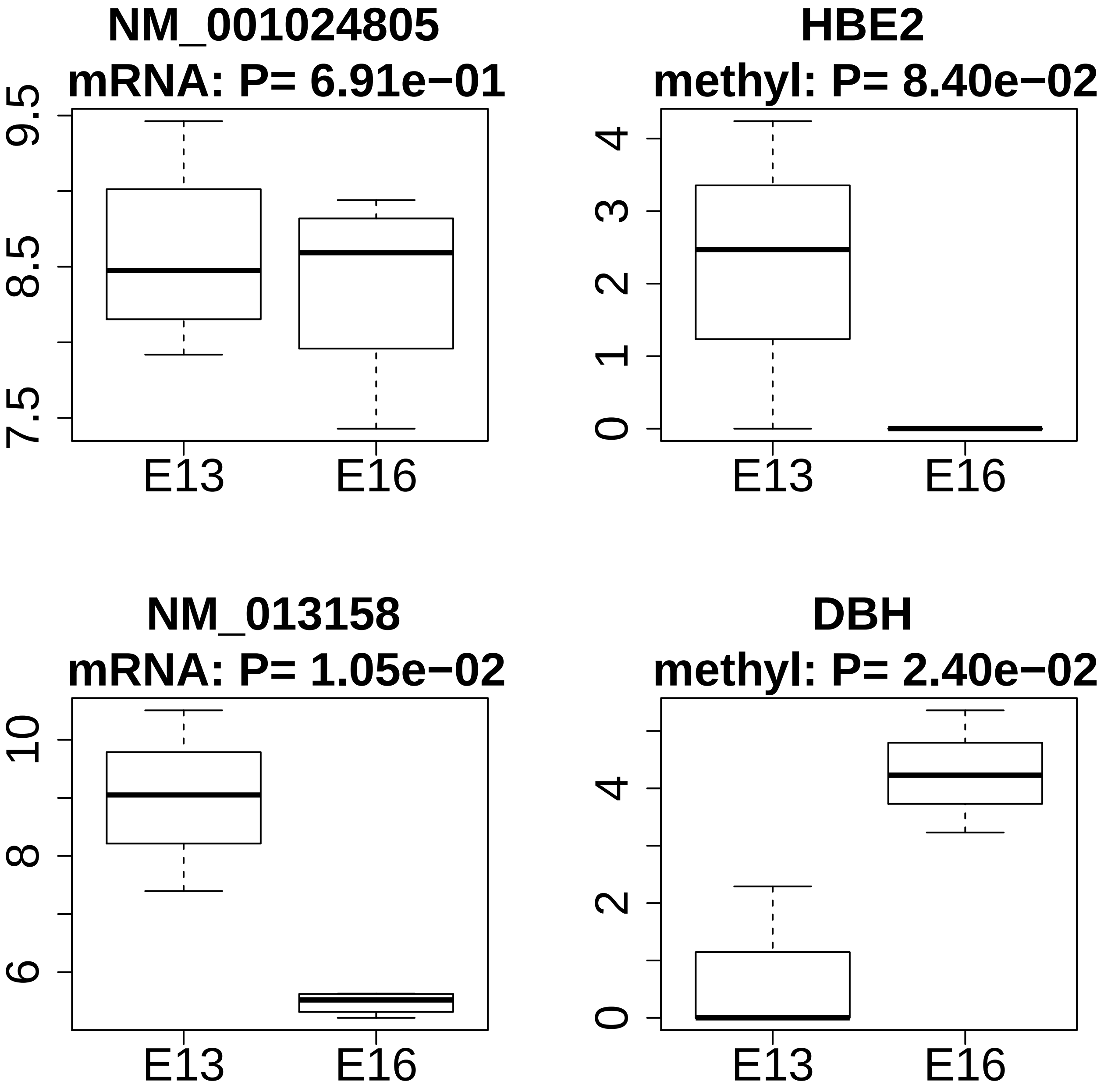
NM_001024805
mRNA: P= 6.91e−01
9.5
8.5
7.5
E13
E16
HBE2
methyl: P= 8.40e−02
4
3
2
1
0
E13
E16
NM_013158
mRNA: P= 1.05e−02
10
8
6
E13
E16
DBH
methyl: P= 2.40e−02
4
2
0
E13
E16

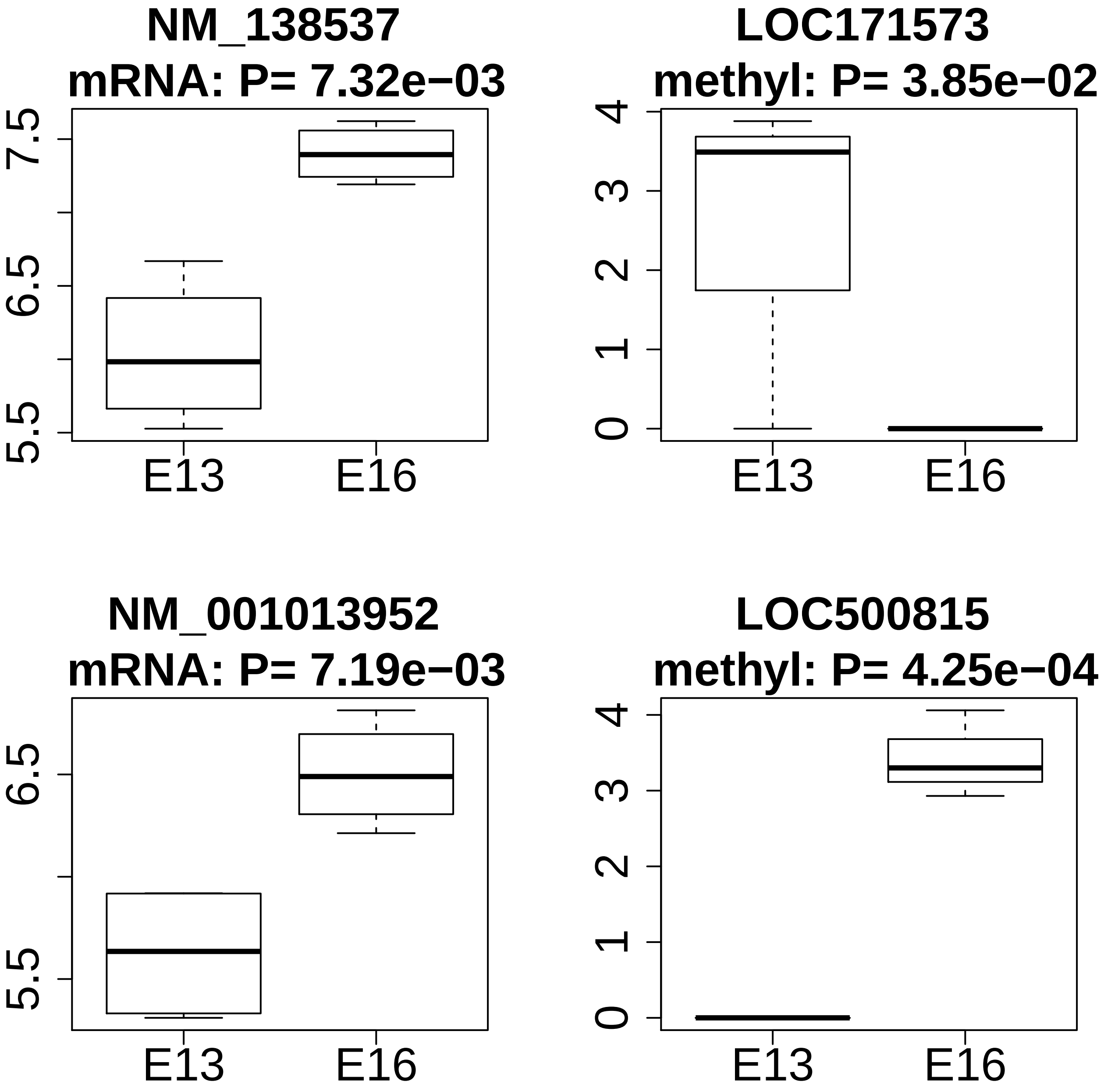
NM_138537
mRNA: P= 7.32e−03
5.5
6.5
7.5
E13
E16
LOC171573
methyl: P= 3.85e−02
0
1
2
3
4
E13
E16
NM_001013952
mRNA: P= 7.19e−03
5.5
6.5
E13
E16
LOC500815
methyl: P= 4.25e−04
0
1
2
3
4
E13
E16

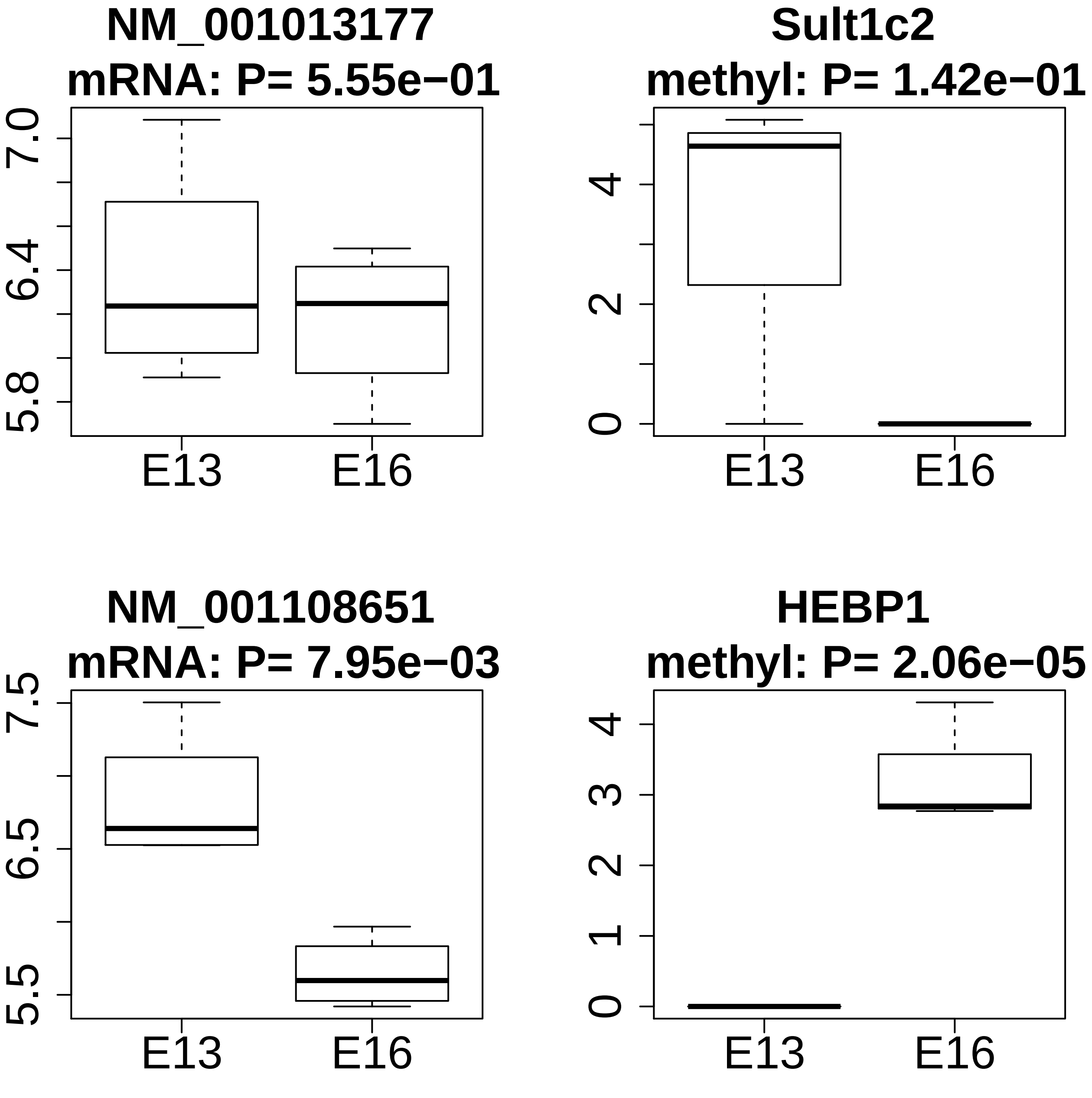
NM_001013177
mRNA: P= 5.55e−01
7.0
6.4
5.8
E13
E16
Sult1c2
methyl: P= 1.42e−01
4
2
0
E13
E16
NM_001108651
mRNA: P= 7.95e−03
7.5
6.5
5.5
E13
E16
HEBP1
methyl: P= 2.06e−05
4
3
2
1
0
E13
E16

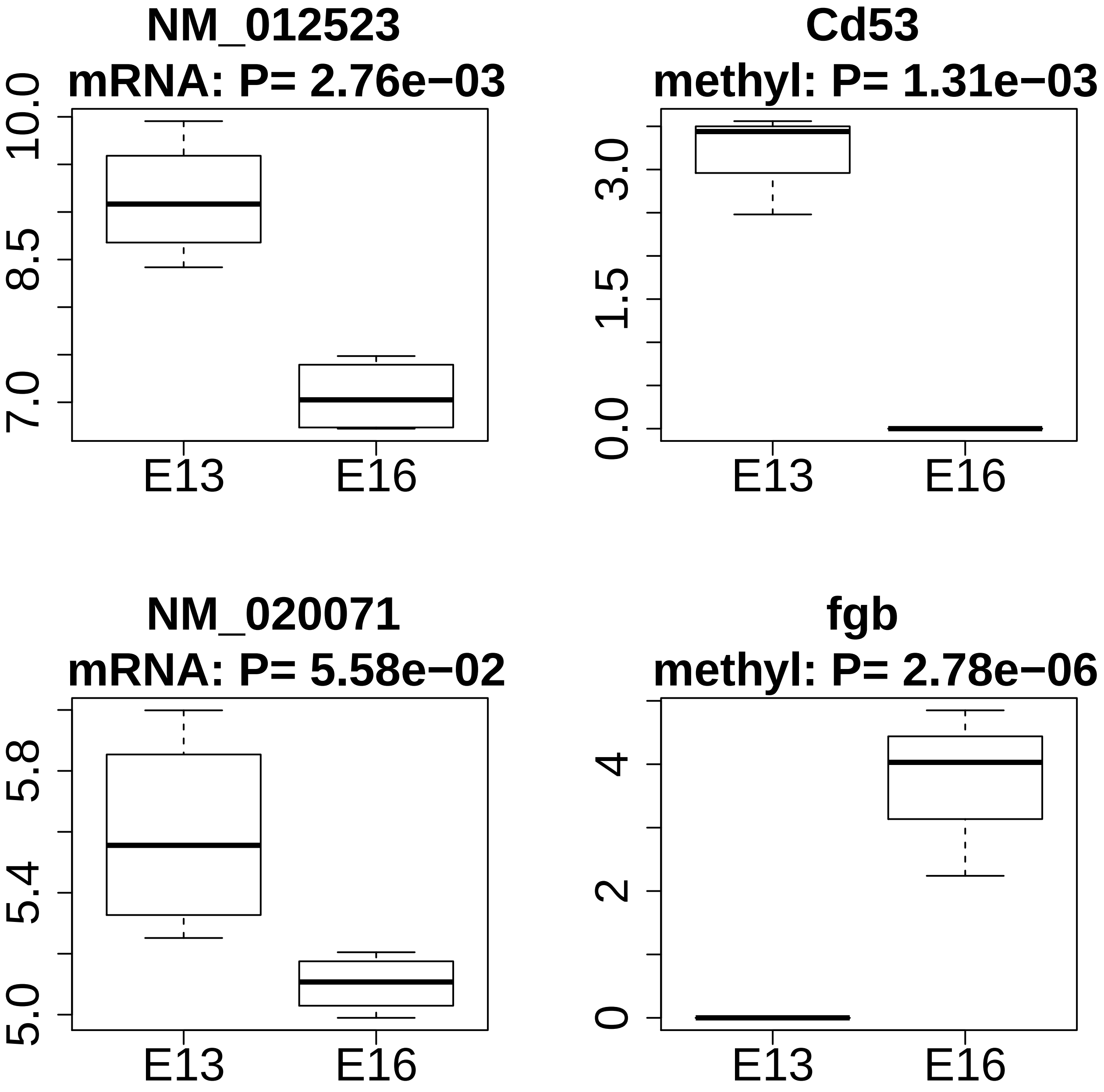

NM_012523
mRNA: P= 2.76e−03
10.0
8.5
7.0
E13
E16
Cd53
methyl: P= 1.31e−03
3.0
1.5
0.0
E13
E16
NM_020071
mRNA: P= 5.58e−02
5.8
5.4
5.0
E13
E16
fgb
methyl: P= 2.78e−06
4
2
0
E13
E16

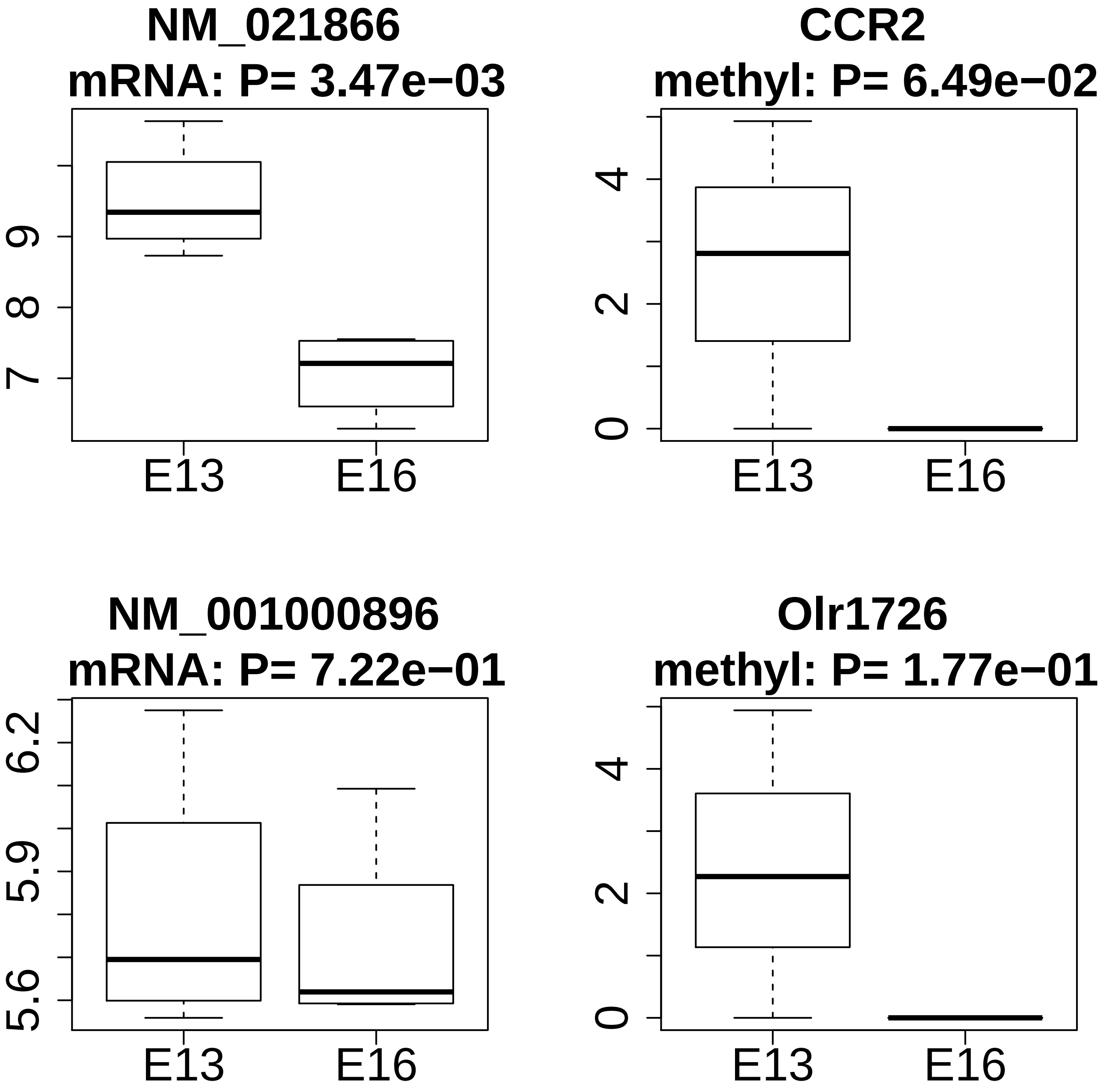
NM_021866
mRNA: P= 3.47e−03
7
8
9
E13
E16
CCR2
methyl: P= 6.49e−02
0
2
4
E13
E16
NM_001000896
mRNA: P= 7.22e−01
5.6
5.9
6.2
E13
E16
Olr1726
methyl: P= 1.77e−01
0
2
4
E13
E16

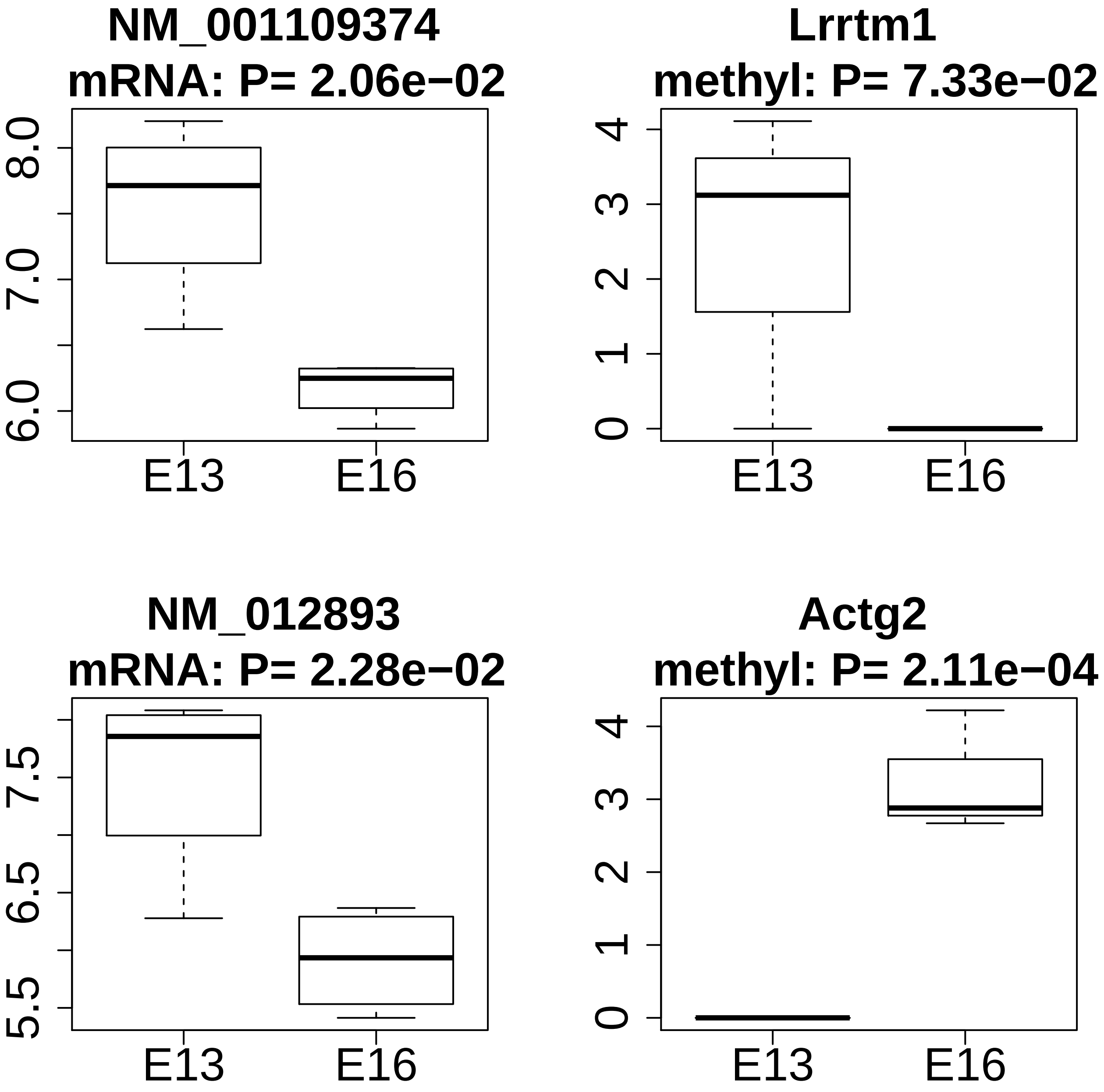
NM_001109374
mRNA: P= 2.06e−02
8.0
7.0
6.0
E13
E16
Lrrtm1
methyl: P= 7.33e−02
4
3
2
1
0
E13
E16
NM_012893
mRNA: P= 2.28e−02
7.5
6.5
5.5
E13
E16
Actg2
methyl: P= 2.11e−04
4
3
2
1
0
E13
E16

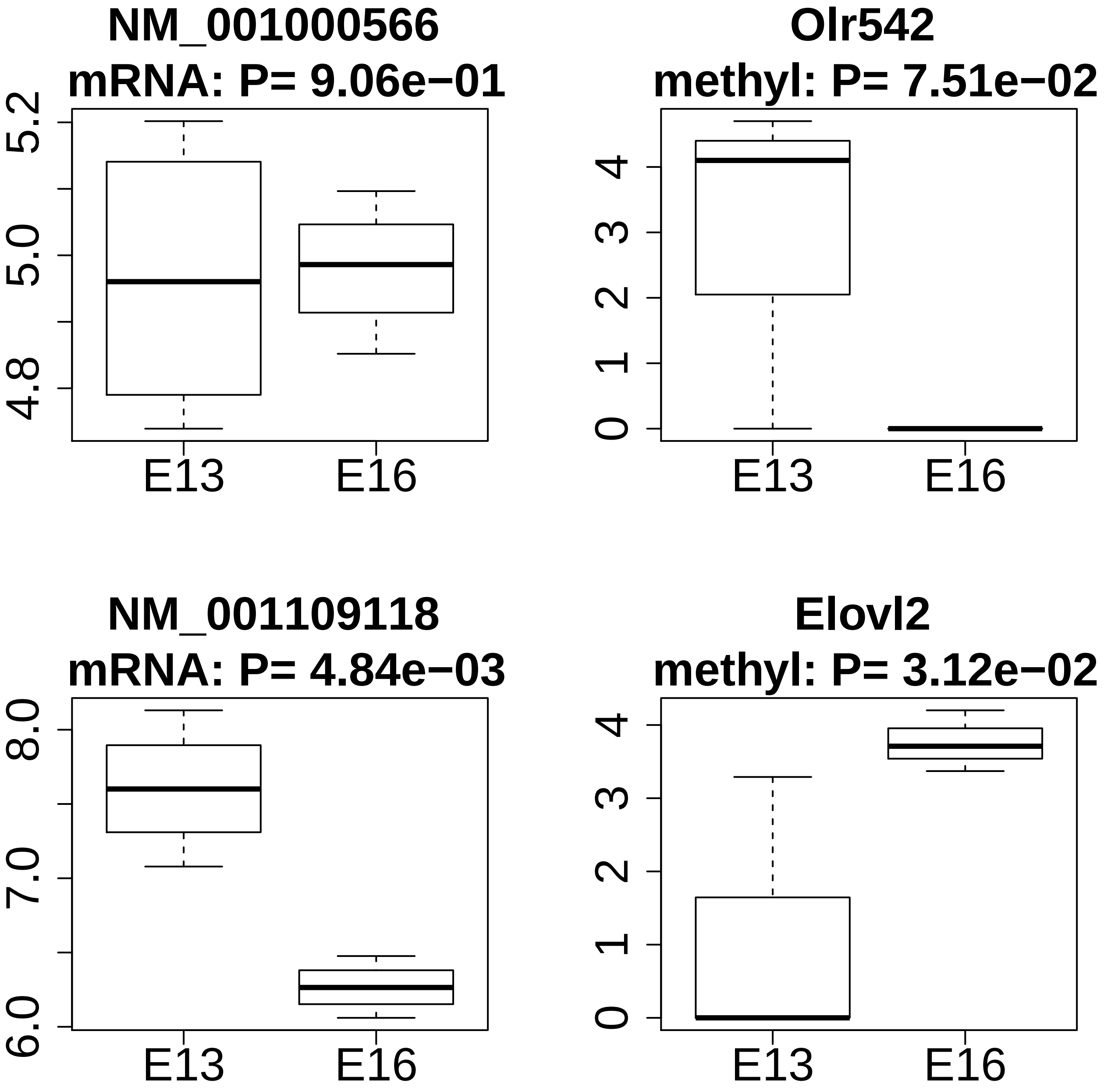
NM_001000566
mRNA: P= 9.06e−01
5.2
5.0
4.8
E13
E16
Olr542
methyl: P= 7.51e−02
4
3
2
1
0
E13
E16
NM_001109118
mRNA: P= 4.84e−03
8.0
7.0
6.0
E13
E16
Elovl2
methyl: P= 3.12e−02
4
3
2
1
0
E13
E16

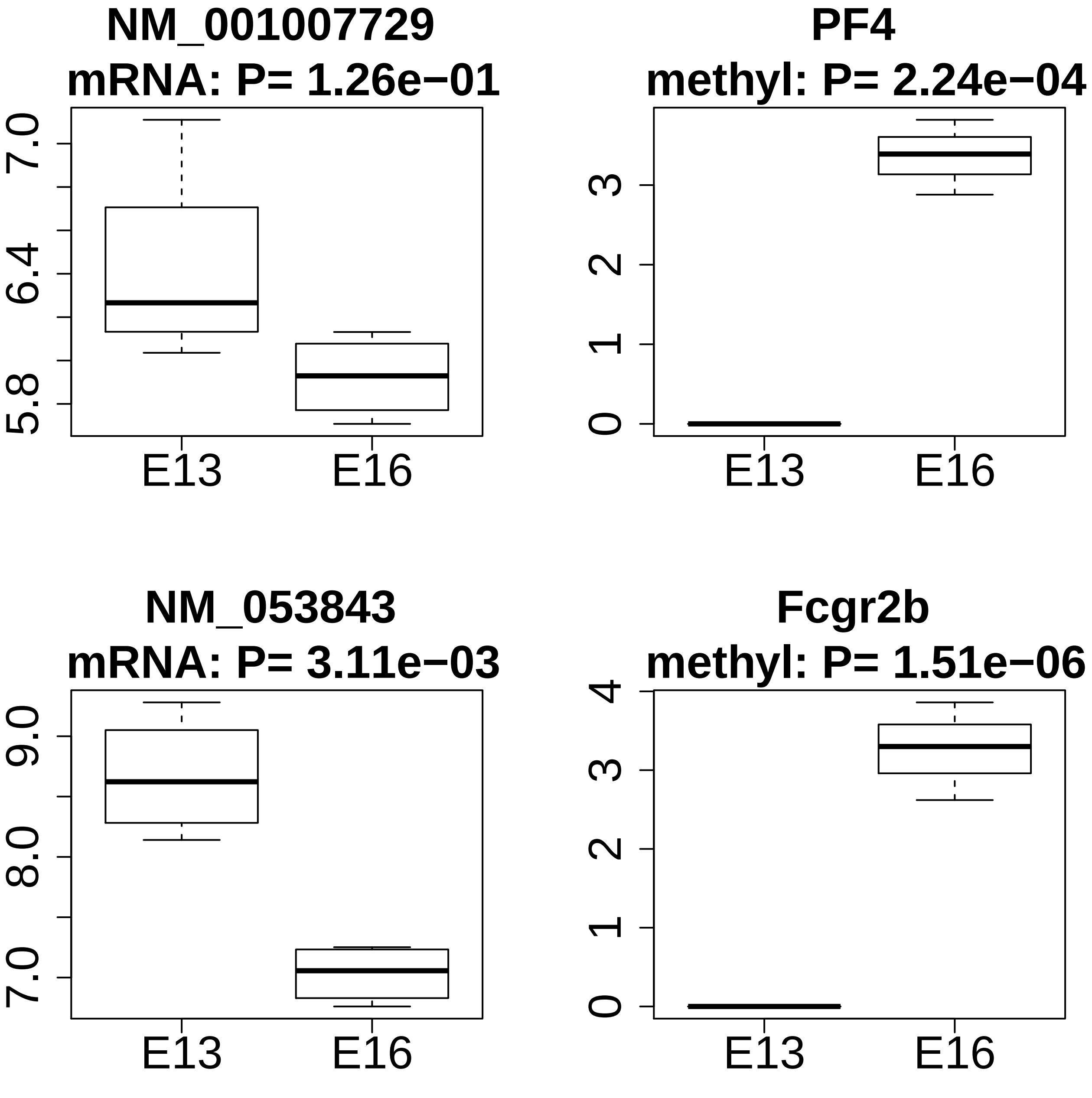
NM_001007729
mRNA: P= 1.26e−01
7.0
6.4
5.8
E13
E16
PF4
methyl: P= 2.24e−04
0
1
2
3
E13
E16
NM_053843
mRNA: P= 3.11e−03
9.0
8.0
7.0
E13
E16
Fcgr2b
methyl: P= 1.51e−06
0
1
2
3
4
E13
E16

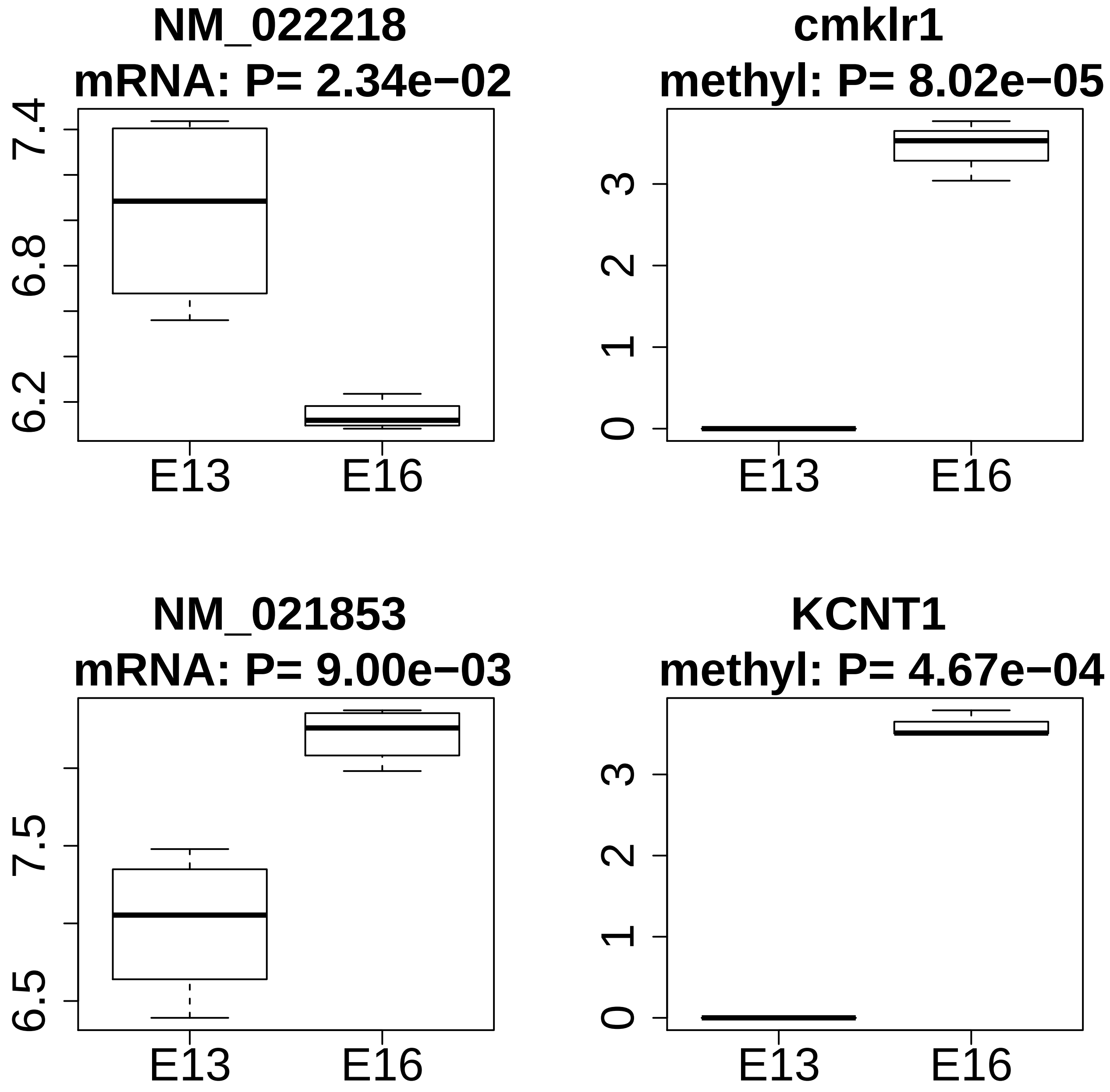

NM_022218
mRNA: P= 2.34e−02
7.4
6.8
6.2
E13
E16
cmklr1
methyl: P= 8.02e−05
3
2
1
0
E13
E16
NM_021853
mRNA: P= 9.00e−03
7.5
6.5
E13
E16
KCNT1
methyl: P= 4.67e−04
3
2
1
0
E13
E16

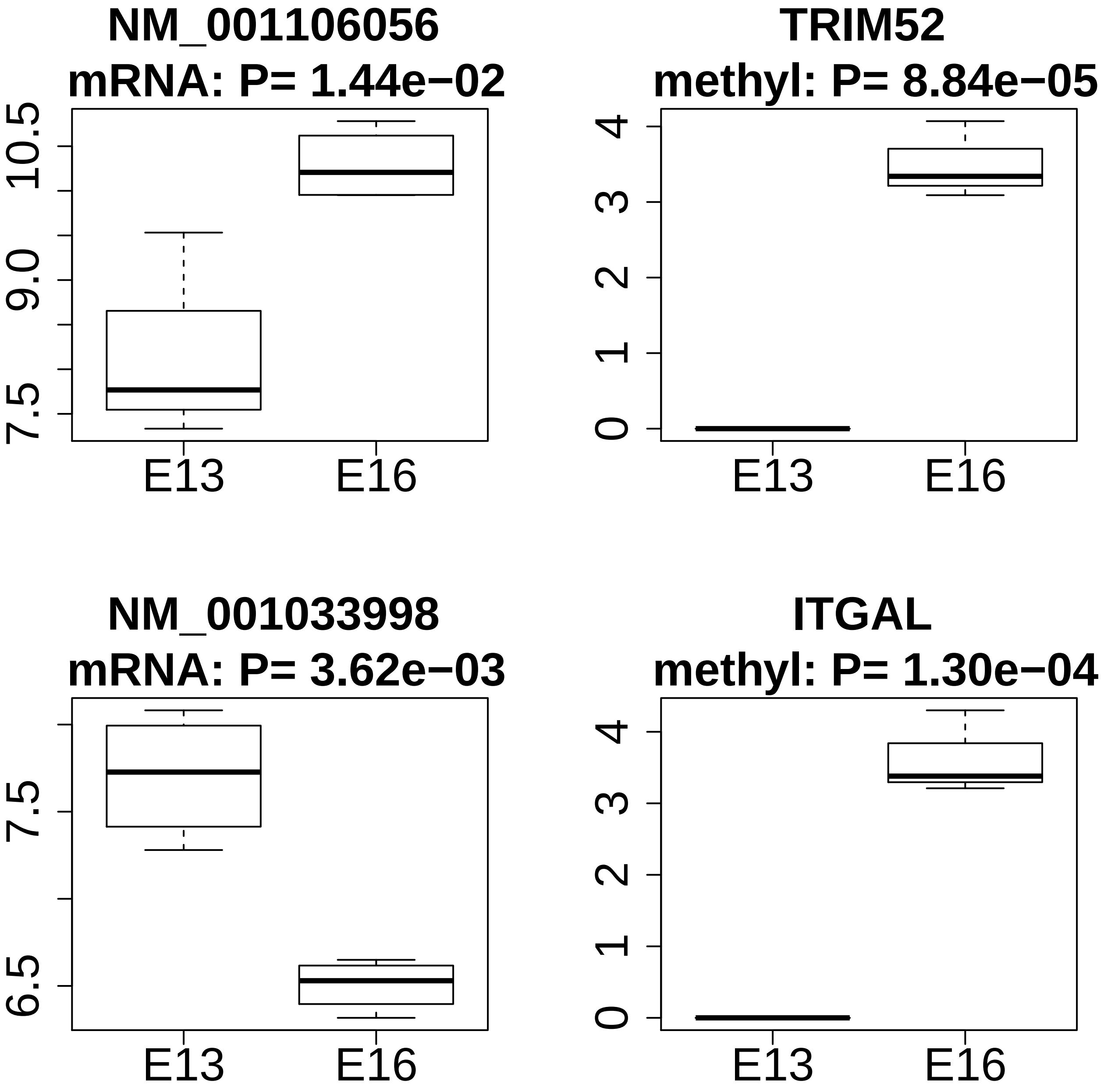
NM_001106056
mRNA: P= 1.44e−02
7.5
9.0
10.5
E13
E16
TRIM52
methyl: P= 8.84e−05
0
1
2
3
4
E13
E16
NM_001033998
mRNA: P= 3.62e−03
6.5
7.5
E13
E16
ITGAL
methyl: P= 1.30e−04
0
1
2
3
4
E13
E16

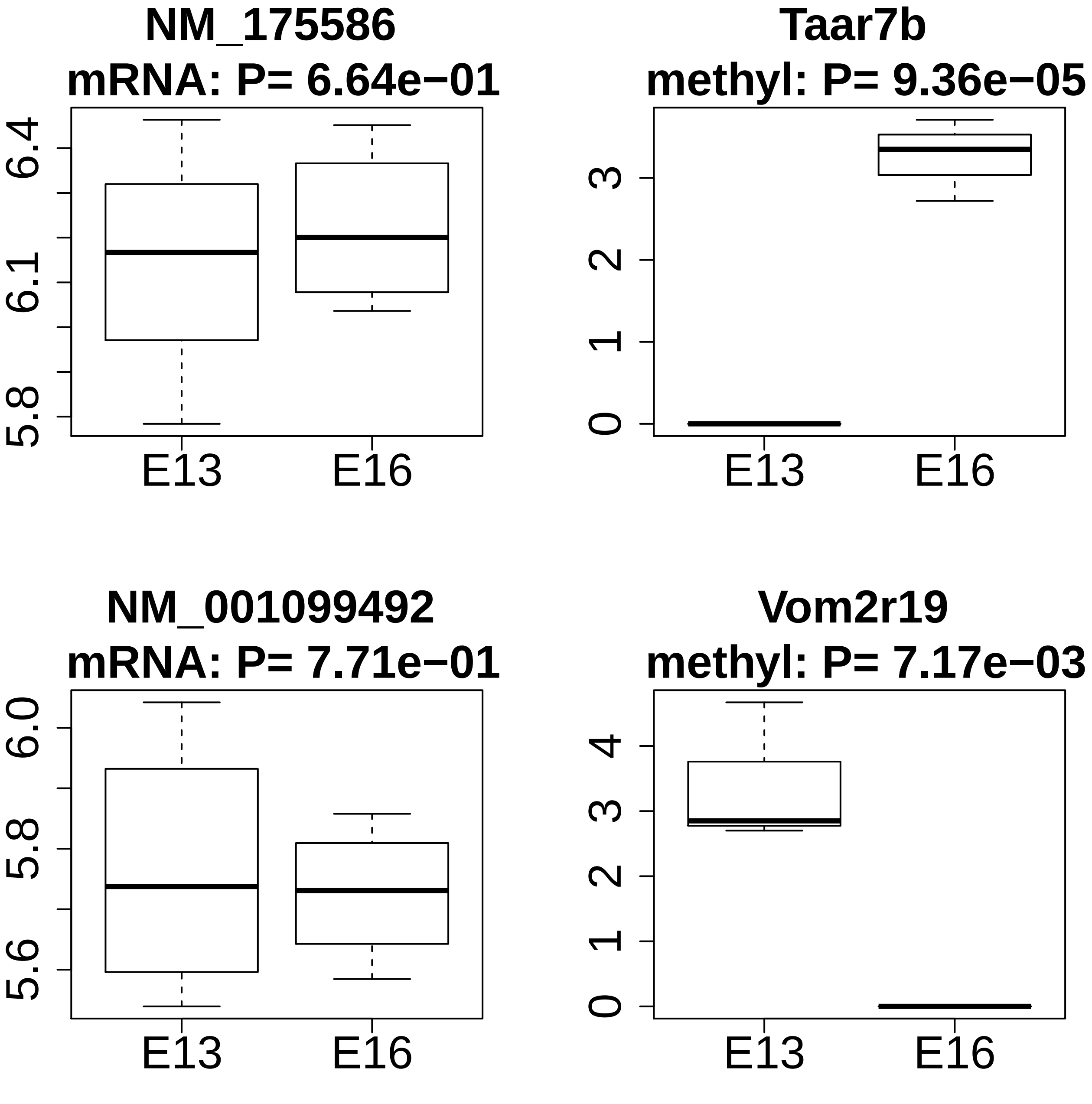

NM_175586
mRNA: P= 6.64e−01
5.8
6.1
6.4
E13
E16
Taar7b
methyl: P= 9.36e−05
0
1
2
3
E13
E16
NM_001099492
mRNA: P= 7.71e−01
5.6
5.8
6.0
E13
E16
Vom2r19
methyl: P= 7.17e−03
0
1
2
3
4
E13
E16

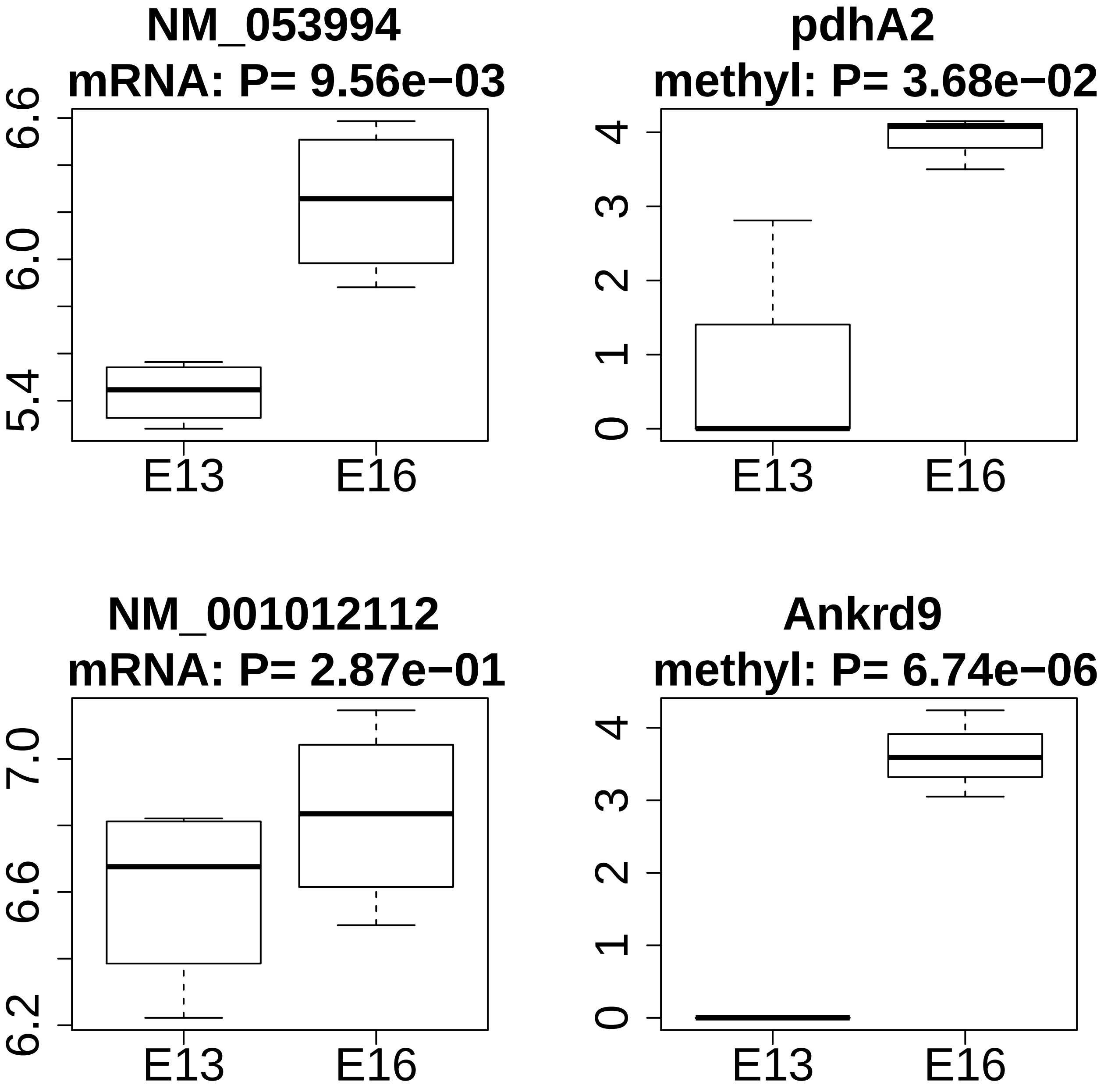
NM_053994
mRNA: P= 9.56e−03
5.4
6.0
6.6
E13
E16
pdhA2
methyl: P= 3.68e−02
0
1
2
3
4
E13
E16
NM_001012112
mRNA: P= 2.87e−01
6.2
6.6
7.0
E13
E16
Ankrd9
methyl: P= 6.74e−06
0
1
2
3
4
E13
E16

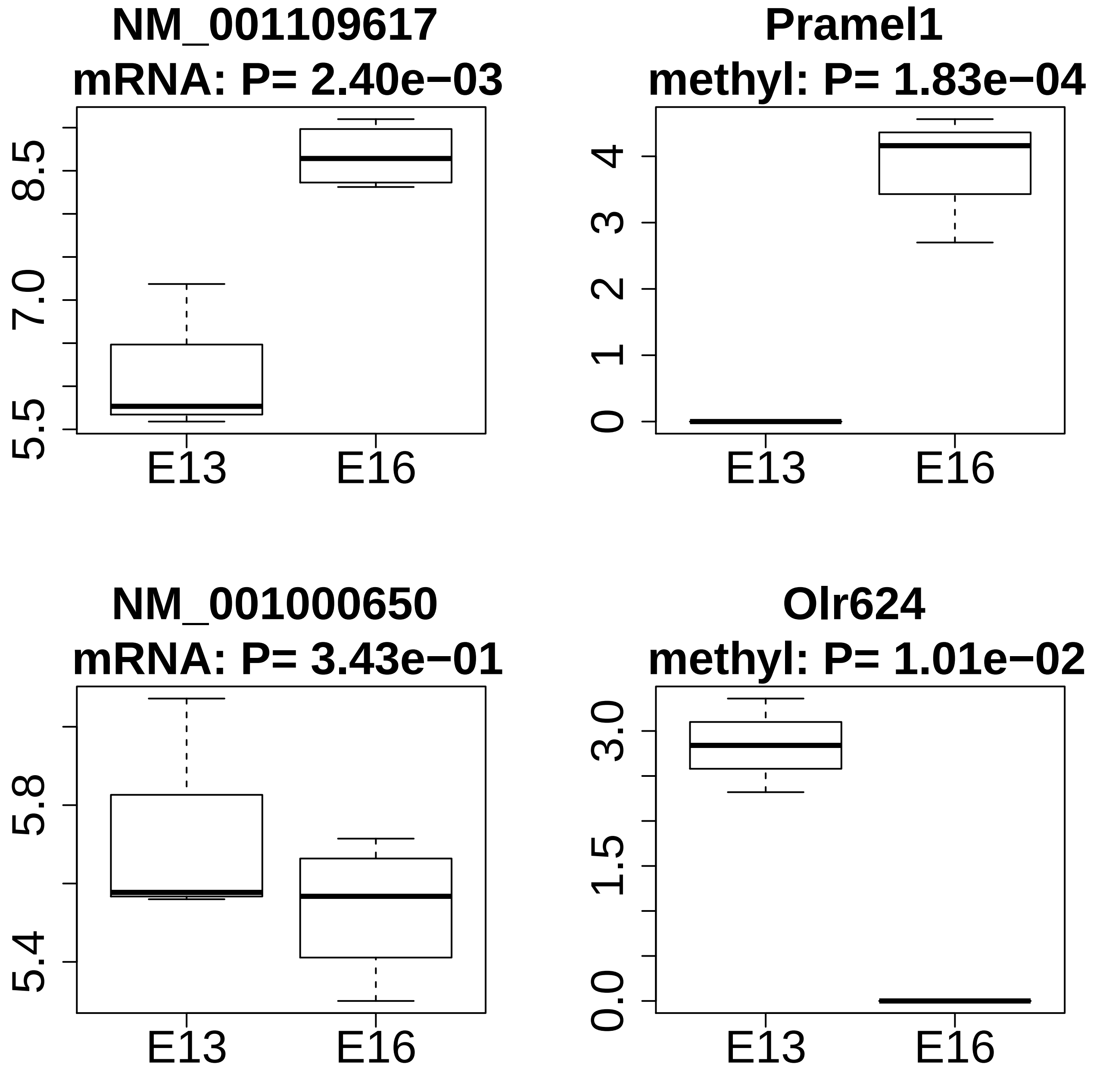
NM_001109617
mRNA: P= 2.40e−03
5.5
7.0
8.5
E13
E16
Pramel1
methyl: P= 1.83e−04
0
1
2
3
4
E13
E16
NM_001000650
mRNA: P= 3.43e−01
5.4
5.8
E13
E16
Olr624
methyl: P= 1.01e−02
0.0
1.5
3.0
E13
E16

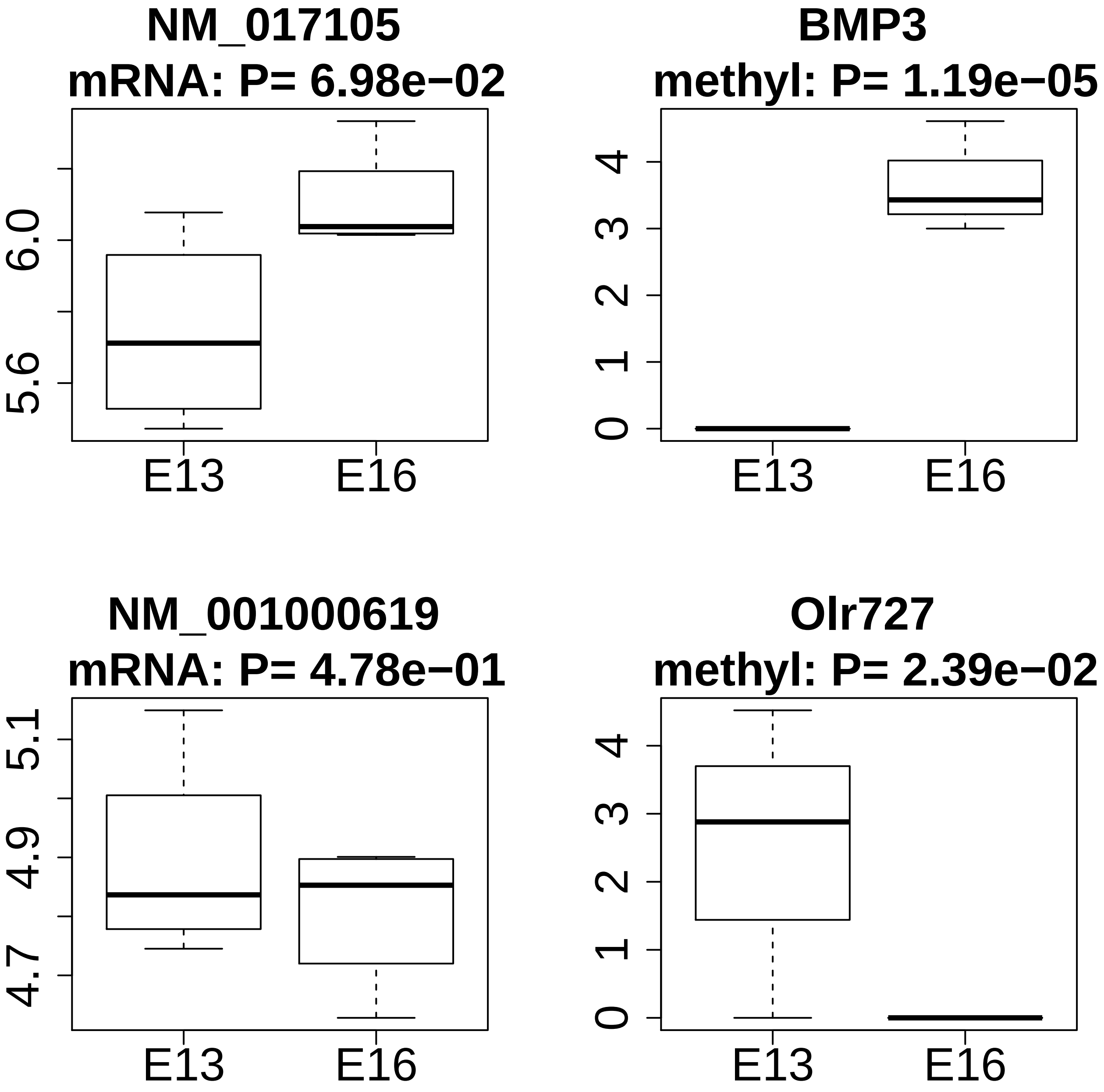
NM_017105
mRNA: P= 6.98e−02
5.6
6.0
E13
E16
BMP3
methyl: P= 1.19e−05
0
1
2
3
4
E13
E16
NM_001000619
mRNA: P= 4.78e−01
4.7
4.9
5.1
E13
E16
Olr727
methyl: P= 2.39e−02
0
1
2
3
4
E13
E16

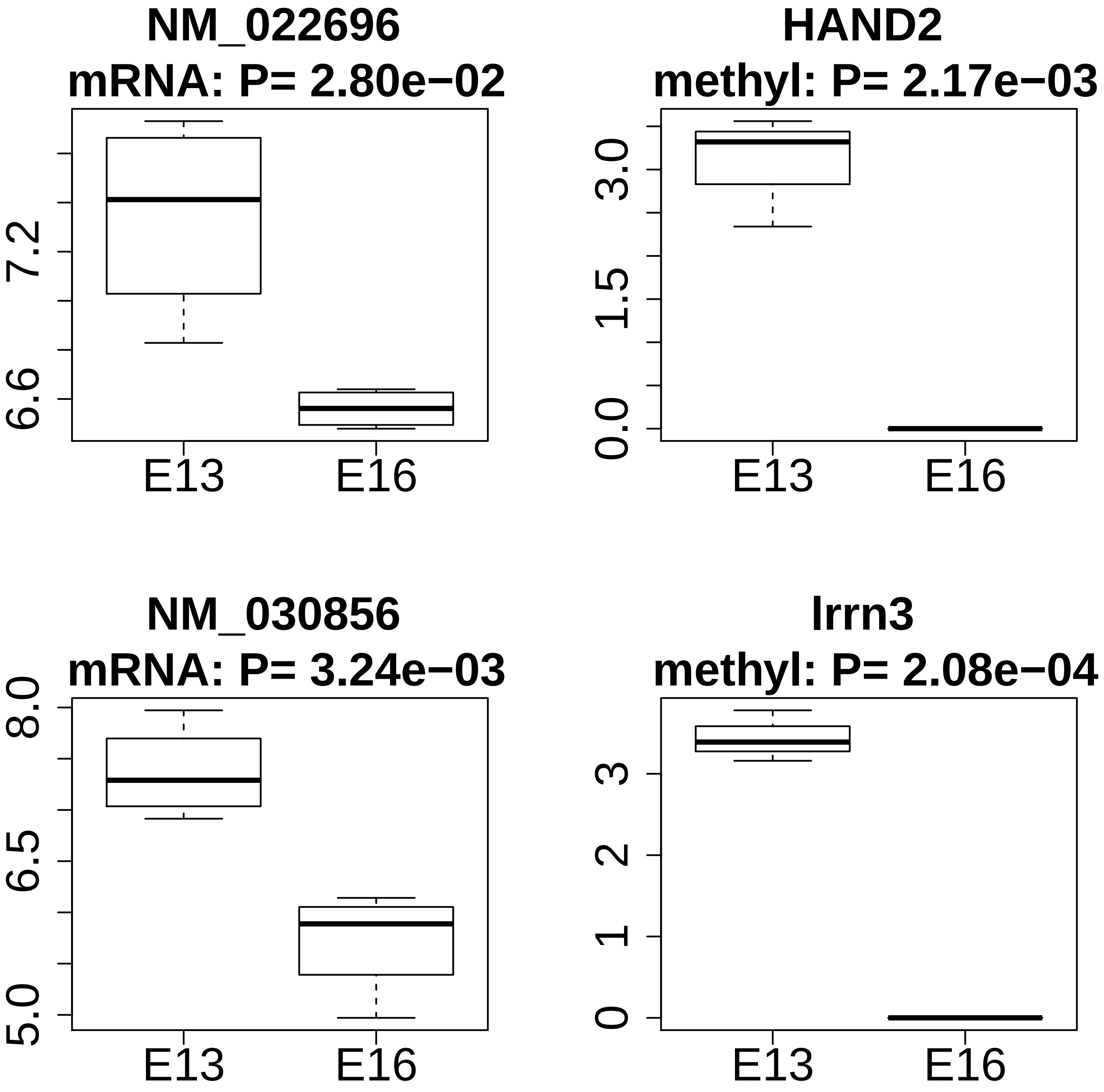
NM_022696
mRNA: P= 2.80e−02
6.6
7.2
E13
E16
HAND2
methyl: P= 2.17e−03
0.0
1.5
3.0
E13
E16
NM_030856
mRNA: P= 3.24e−03
5.0
6.5
8.0
E13
E16
lrrn3
methyl: P= 2.08e−04
0
1
2
3
E13
E16

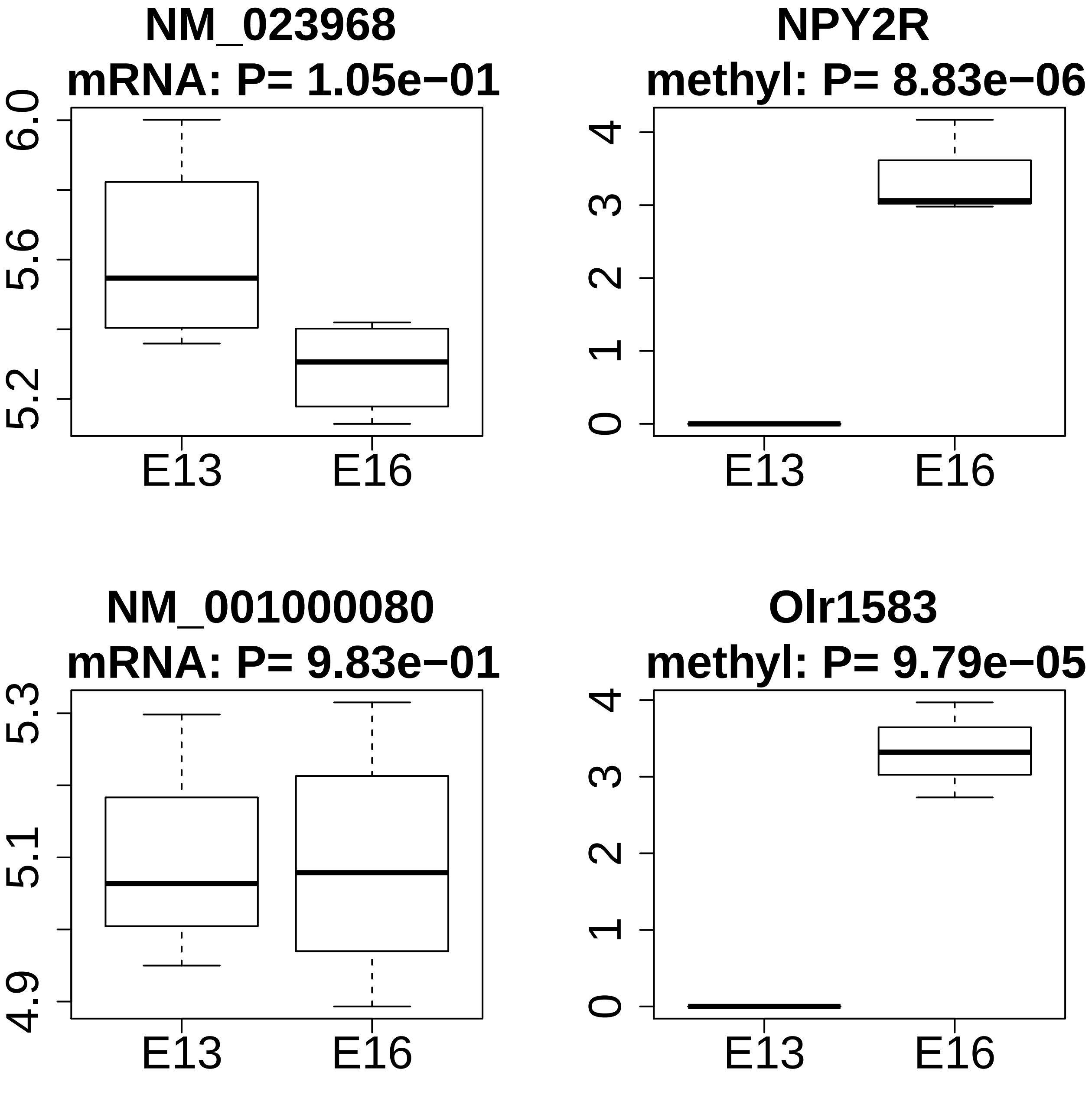
NM_023968
mRNA: P= 1.05e−01
6.0
5.6
5.2
E13
E16
NPY2R
methyl: P= 8.83e−06
4
3
2
1
0
E13
E16
NM_001000080
mRNA: P= 9.83e−01
5.3
5.1
4.9
E13
E16
Olr1583
methyl: P= 9.79e−05
4
3
2
1
0
E13
E16

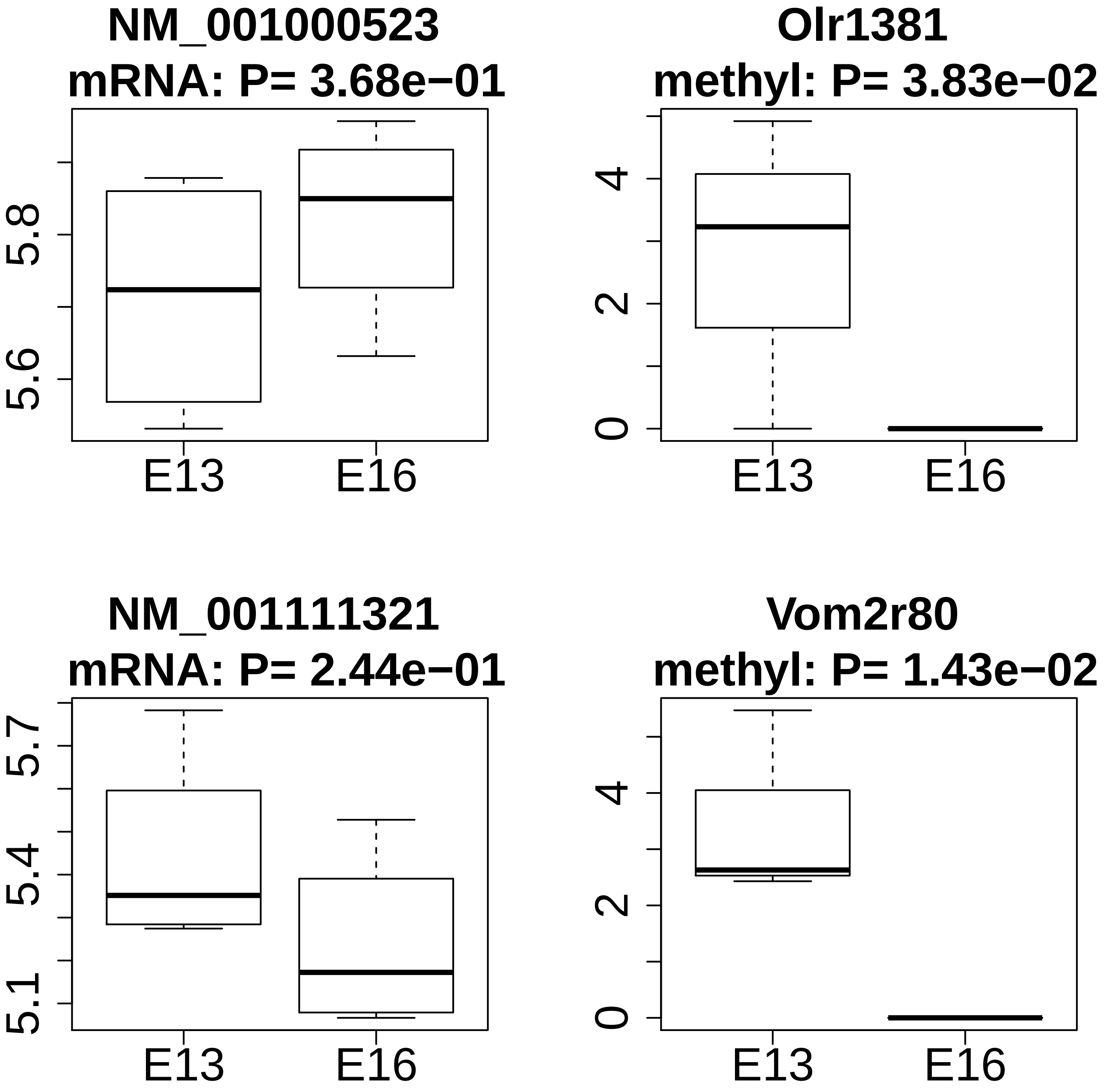

NM_001000523
mRNA: P= 3.68e−01
5.6
5.8
E13
E16
Olr1381
methyl: P= 3.83e−02
0
2
4
E13
E16
NM_001111321
mRNA: P= 2.44e−01
5.1
5.4
5.7
E13
E16
Vom2r80
methyl: P= 1.43e−02
0
2
4
E13
E16

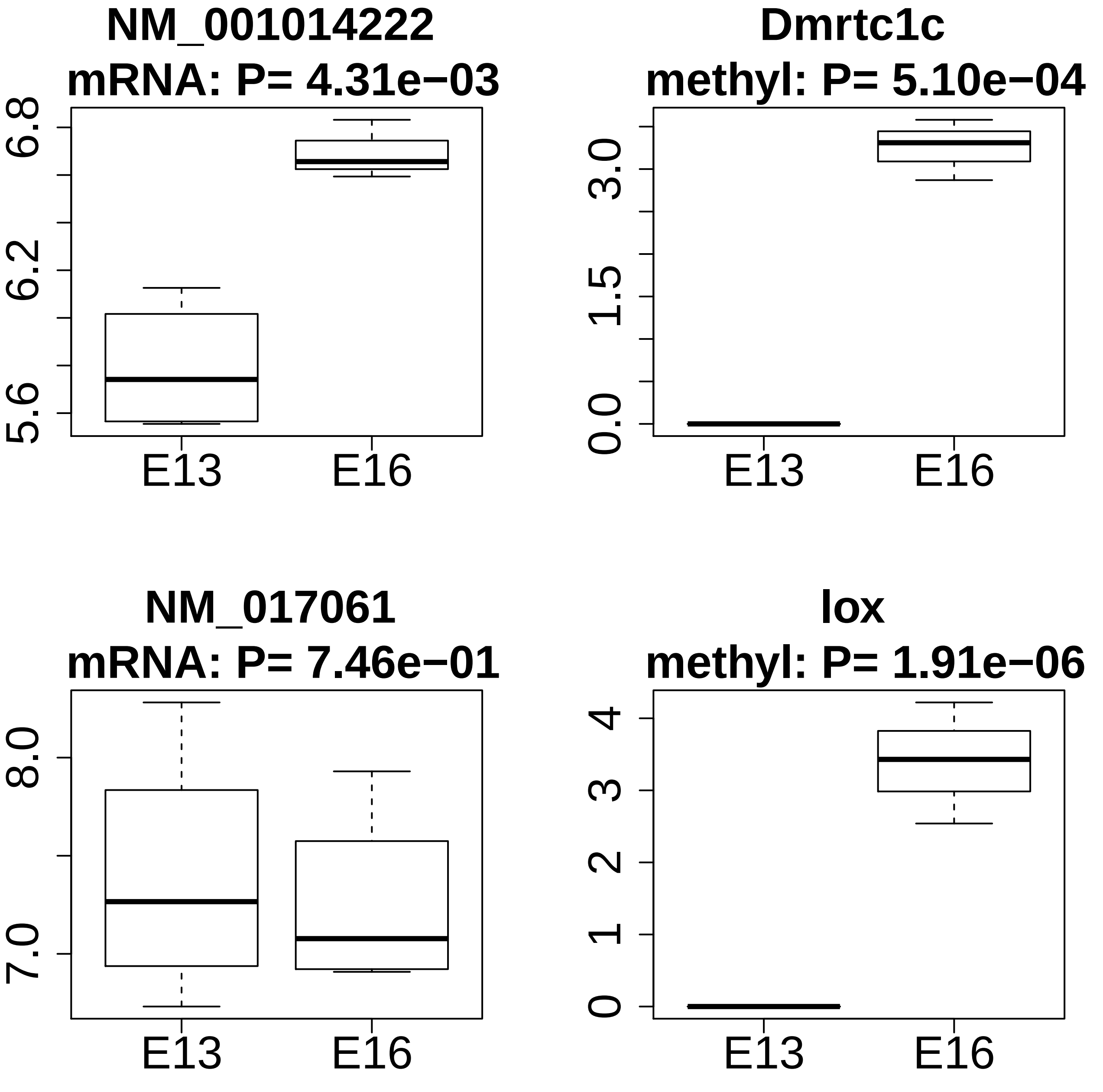
NM_001014222
mRNA: P= 4.31e−03
5.6
6.2
6.8
E13
E16
Dmrtc1c
methyl: P= 5.10e−04
0.0
1.5
3.0
E13
E16
NM_017061
mRNA: P= 7.46e−01
7.0
8.0
E13
E16
lox
methyl: P= 1.91e−06
0
1
2
3
4
E13
E16

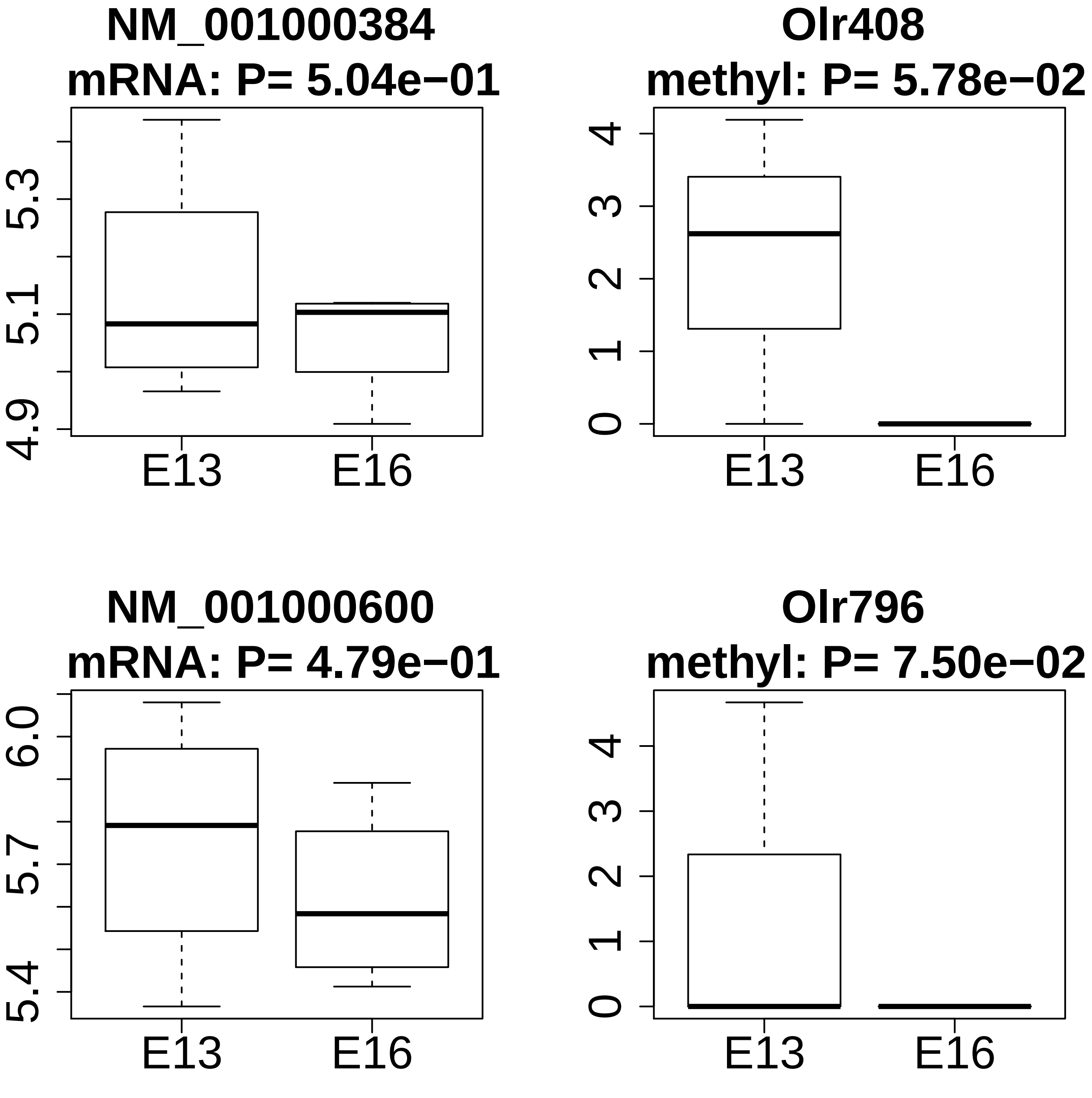
NM_001000384
mRNA: P= 5.04e−01
4.9
5.1
5.3
E13
E16
Olr408
methyl: P= 5.78e−02
0
1
2
3
4
E13
E16
NM_001000600
mRNA: P= 4.79e−01
5.4
5.7
6.0
E13
E16
Olr796
methyl: P= 7.50e−02
0
1
2
3
4
E13
E16

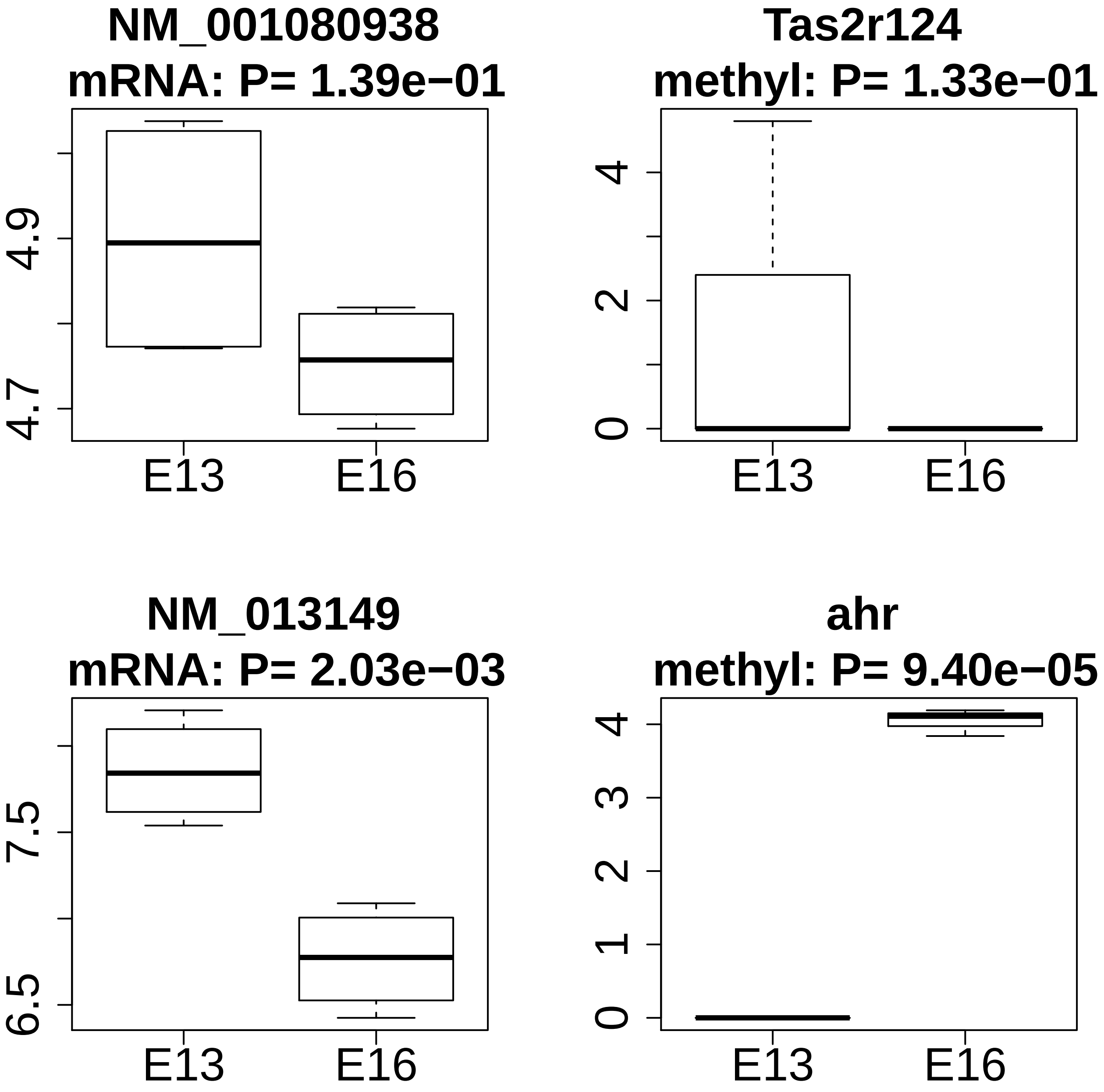

NM_001080938
mRNA: P= 1.39e−01
4.7
4.9
E13
E16
Tas2r124
methyl: P= 1.33e−01
0
2
4
E13
E16
NM_013149
mRNA: P= 2.03e−03
6.5
7.5
E13
E16
ahr
methyl: P= 9.40e−05
0
1
2
3
4
E13
E16

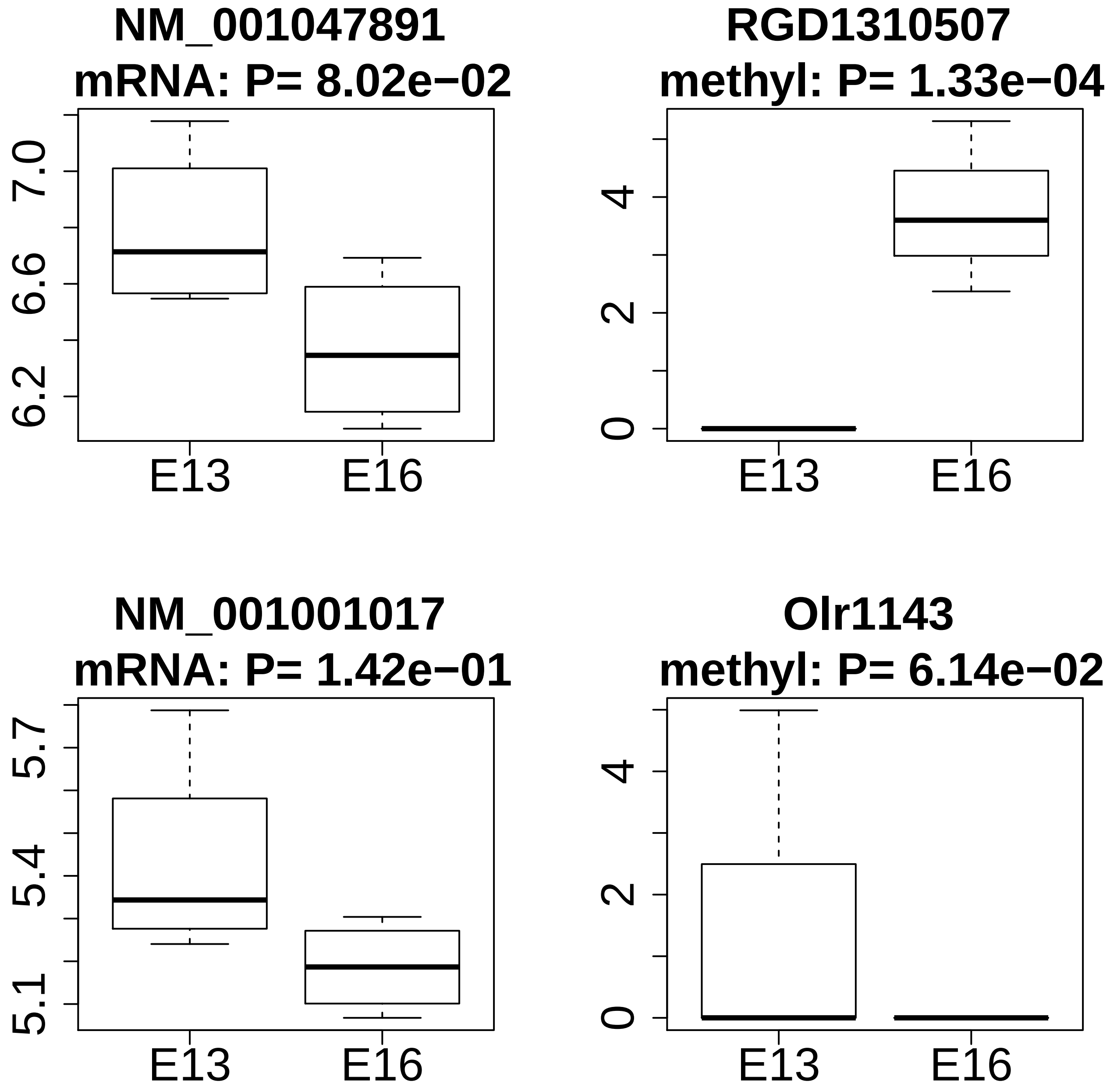

NM_001047891
mRNA: P= 8.02e−02
6.2
6.6
7.0
E13
E16
RGD1310507
methyl: P= 1.33e−04
0
2
4
E13
E16
NM_001001017
mRNA: P= 1.42e−01
5.1
5.4
5.7
E13
E16
Olr1143
methyl: P= 6.14e−02
0
2
4
E13
E16

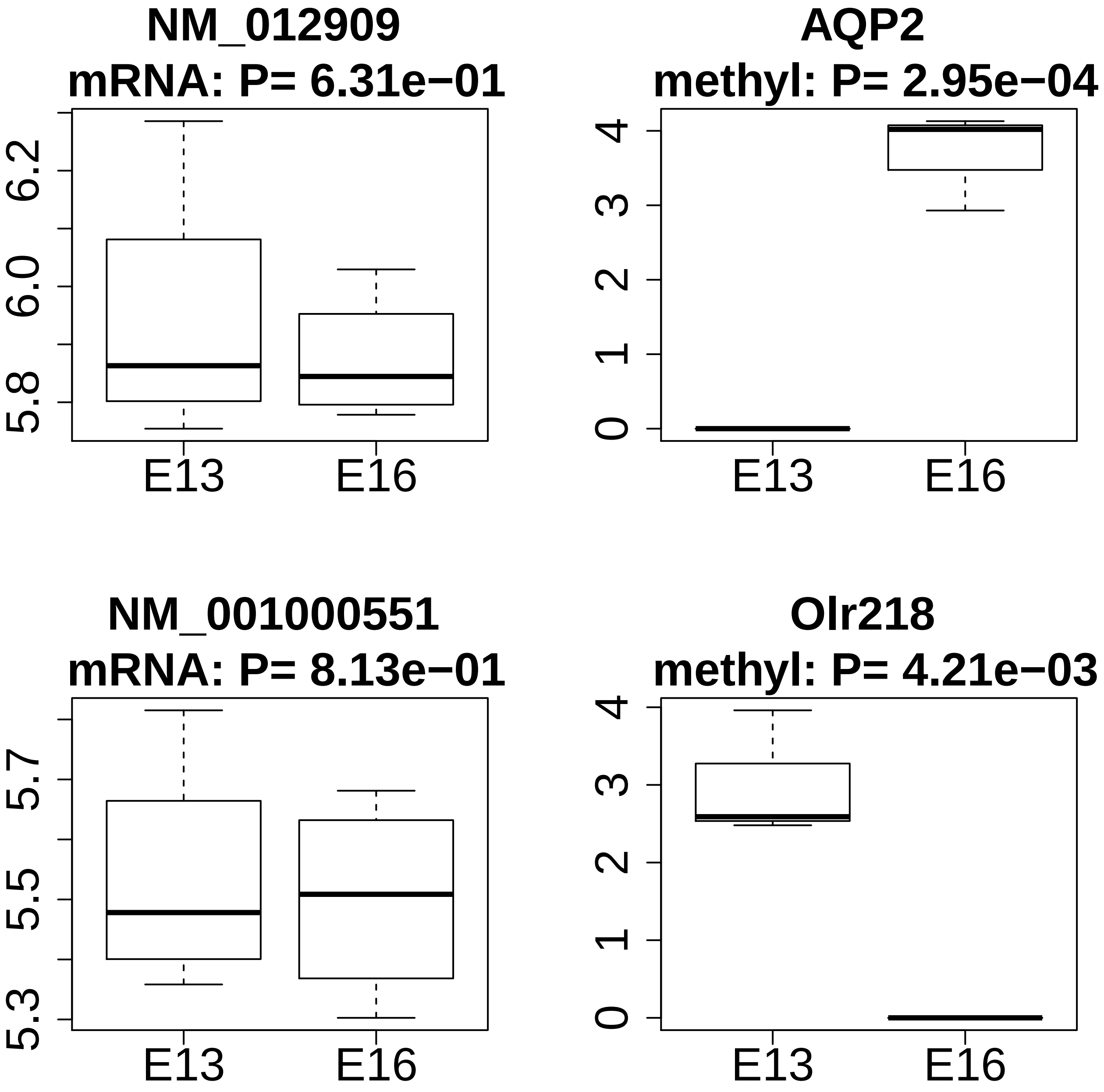
NM_012909
mRNA: P= 6.31e−01
6.2
6.0
5.8
E13
E16
AQP2
methyl: P= 2.95e−04
4
3
2
1
0
E13
E16
NM_001000551
mRNA: P= 8.13e−01
5.7
5.5
5.3
E13
E16
Olr218
methyl: P= 4.21e−03
4
3
2
1
0
E13
E16

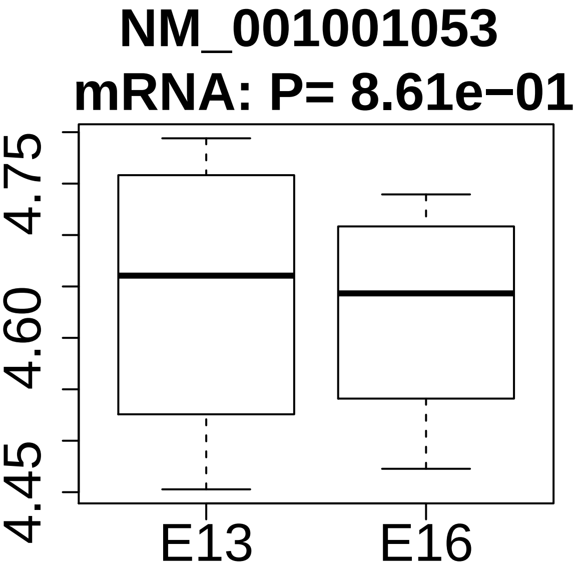
NM_001001053
mRNA: P= 8.61e−01
4.45
4.60
4.75
E13
E16

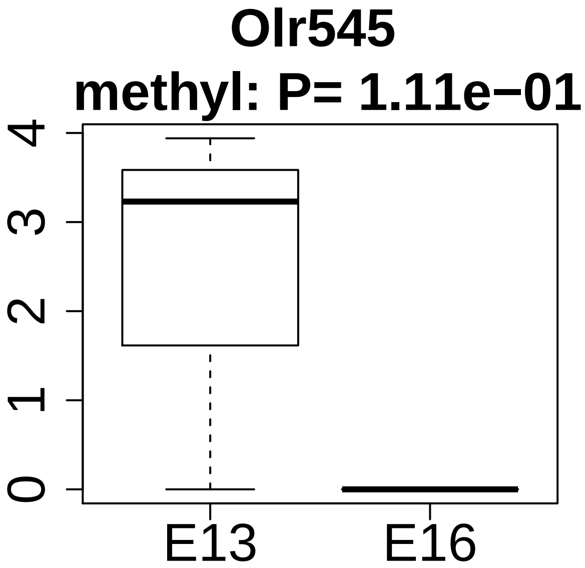
Olr545
methyl: P= 1.11e−01
0
1
2
3
4
E13
E16
