# Supplementary figures and images for: Identification of aberrant gene expression associated with aberrant promoter methylation in primordial germ cells between E13 and E16 rat F3 generation vinclozolin lineage
Source: BMC Bioinformatics. 2015 Dec 9;16(Suppl 18):S16. doi: 10.1186/1471-2105-16-S18-S16 (PMC4682393; doi:10.1186/1471-2105-16-S18-S16)

$t$  test based FE

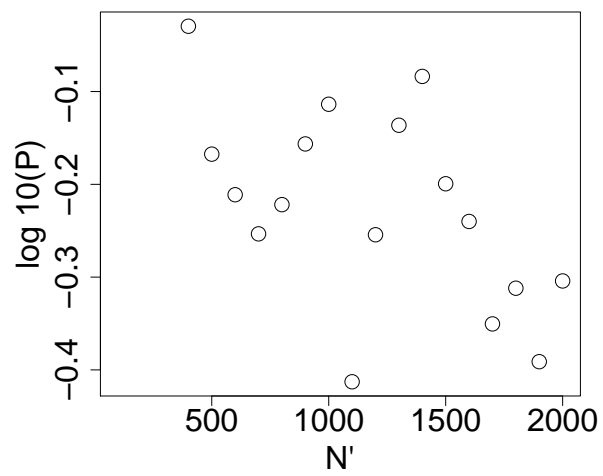

limma based FE

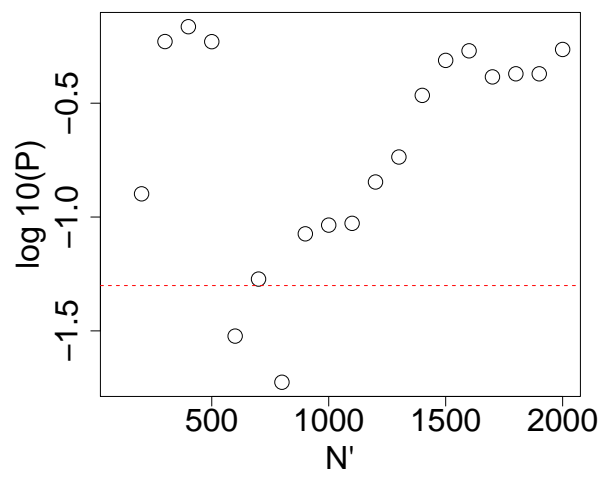

# SAM based FE

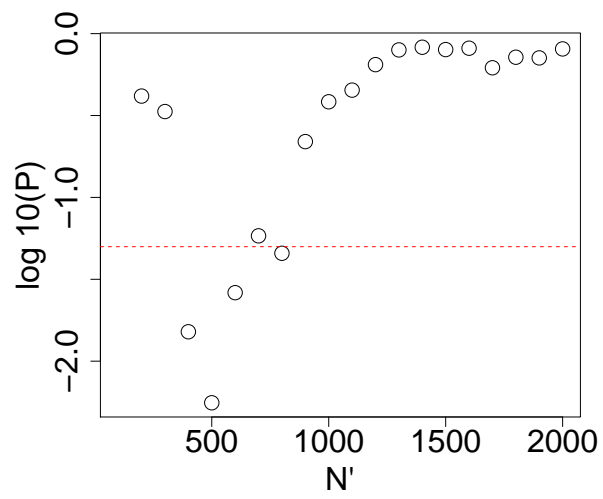

Supplement: Additional file 4 — Dependence of P values upon N' when genes are selected by the t test, limma and SAM instead of PCA-based unsupervised FE. [file 1471-2105-16-S18-S16-S4.pdf]
